# Protein expression of the gp78 E3 ligase predicts poor breast cancer outcome based on race

**DOI:** 10.1172/jci.insight.157465

**Published:** 2022-07-08

**Authors:** Sandeep K. Singhal, Jung S. Byun, Tingfen Yan, Ryan Yancey, Ambar Caban, Sara Gil Hernandez, Sediqua Bufford, Stephen M. Hewitt, Joy Winfield, Jaya Pradhan, Vesco Mustkov, Jasmine A. McDonald, Eliseo J. Pérez-Stable, Anna María Nápoles, Nasreen Vohra, Adriana De Siervi, Clayton Yates, Melissa B. Davis, Mei Yang, Yien Che Tsai, Allan M. Weissman, Kevin Gardner

**Affiliations:** 1 Department of Pathology, School of Medicine and Health Sciences,; 2 Department of Biomedical Engineering, School of Electrical Engineering and Computer Science, University of North Dakota, Grand Forks, North Dakota, USA.; 3 Division of Intramural Research, National Institutes of Minority Health and Health Disparities, NIH, Bethesda, Maryland, USA.; 4 Department of Pathology and Cell Biology, Columbia University Irvine Medical Center, New York, New York, USA.; 5 Masters of Science Biotechnology, Morehouse School of Medicine, Atlanta, Georgia, USA.; 6 Laboratory of Pathology, Centers for Cancer Research, National Cancer Institute, NIH, Bethesda, Maryland, USA.; 7 Department of Epidemiology, Mailman School of Public Health, Columbia University Irving Medical Center, New York, New York, USA.; 8 Brody School of Medicine, East Carolina University, Greenville, North Carolina, USA.; 9 Laboratory of Molecular Oncology and New Therapeutic Targets, Institute of Biology and Experimental Medicine (IBYME), CONICET, Argentina; 10 Department of Biology and Center for Cancer Research, Tuskegee University, Tuskegee, Alabama, USA.; 11 Weill Cornell Medicine, New York, New York, USA; 12 Laboratory of Protein Dynamics and Signaling, Center for Cancer Research, National Cancer Institute, Frederick, Maryland, USA.

**Keywords:** Cell Biology, Oncology, Breast cancer

## Abstract

Women of African ancestry suffer higher rates of breast cancer mortality compared with all other groups in the United States. Though the precise reasons for these disparities remain unclear, many recent studies have implicated a role for differences in tumor biology. Using an epitope-validated antibody against the endoplasmic reticulum–associated E3 ligase, gp78, we show that elevated levels of gp78 in patient breast cancer cells predict poor survival. Moreover, high levels of gp78 are associated with poor outcomes in both ER^+^ and ER^–^ tumors, and breast cancers expressing elevated amounts of gp78 protein are enriched in gene expression pathways that influence cell cycle, metabolism, receptor-mediated signaling, and cell stress response pathways. In multivariate analysis adjusted for subtype and grade, gp78 protein is an independent predictor of poor outcomes in women of African ancestry. Furthermore, gene expression signatures, derived from patients stratified by gp78 protein expression, are strong predictors of recurrence and pathological complete response in retrospective clinical trial data and share many common features with gene sets previously identified to be overrepresented in breast cancers based on race. These findings implicate a prominent role for gp78 in tumor progression and offer insights into our understanding of racial differences in breast cancer outcomes.

## Graphical abstract

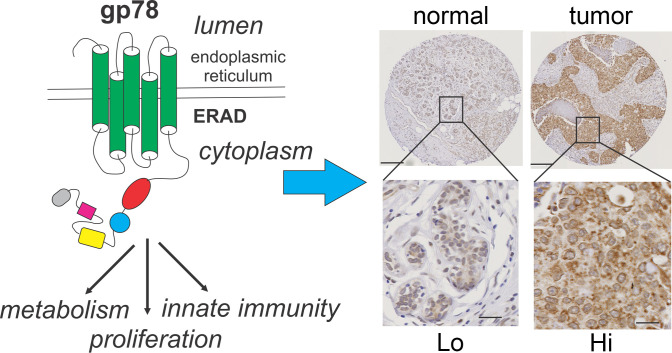

## Introduction

In 2021, breast cancer became the most frequently diagnosed malignancy worldwide (1). This year in the United States, more than 270,000 cases of invasive breast cancer will be newly diagnosed and nearly 40,000 women currently living with a diagnosis of breast cancer will succumb to their disease before the year’s end (2). The burden of this disease, however, is not equally distributed in women of European ancestry compared with African ancestry in the United States. Women of West African ancestry suffer nearly a 40% higher mortality from breast cancer compared with those of European heritage. Though portions of this disparity are rooted in inequities associated with access to health care, aspects of structural racism, and multiple other determinants of health disparities, prevailing evidence suggests a significant role for biology (3–5). This includes aspects of the immune response surrounding the tumor that constitute the tumor microenvironment, in addition to specific attributes of intrinsic tumor biology (6–8). Recent studies have revealed that stratification of outcome risk using available biomarkers, developed with breast cancer cohorts consisting primarily of patients with breast cancer of European ancestry, show significantly lower predictive accuracy in women of African ancestry (6, 9–12). For these reasons, there remains an unmet need to identify, develop, and disseminate new functional biomarkers that will not only provide higher predictive accuracy, but will also reveal insight into breast cancer diagnosis, treatment, and prevention.

The protein gp78 is an endoplasmic reticulum (ER) resident E3 ligase that targets numerous proteins for degradation by ubiquitylation and, through its cytoplasmic E3 ligase domain, plays a major role in the unfolded protein response (UPR) and other adaptive cellular responses to ER stress (13–15). Because of its E3 ligase activity and its residency in the ER, gp78 is believed to exert a major role in facilitating cellular homeostasis in response to the cellular stresses and environmental conditions commonly faced by rapidly growing tumors, including hypoxia, reactive oxygen species, nutrient deprivation, and protein mutation. All of these exposures impact cellular homeostasis through ER stress, subsequent activation of ER-associated degradation (ERAD), and amplification of proteostatic responses (16–22). However, in addition to its participation in ERAD by removing misfolded proteins, a substantial portion of the role played by gp78 in cellular homeostasis can be understood by examining its substrate specificity. Known substrates targeted by the E3 ligase activity of gp78 include the CD3δ T cell receptor subunit (14); the KAI1 tumor suppressor protein (23, 24); apolipoprotein B100 (25); cytochrome P450 proteins CYP3A4, CYP3A5, CYP2A5, CYP2C, and CYP2E1 (26, 27); the homocysteine-induced ER protein (HERP) (28) that stabilizes TKB1 during antiviral responses (29); the viral DNA response adaptor protein, stimulator of IFN genes (STING) (30); the mitofusin mitochondrial fusion regulators MFN1 and MFN2 (31); mutant α-1-antitrypsin (32); the sterol regulator insulin stimulated gene 1 (Insig-1) (33); HMG CoA reductase (34); the diacylglycerol acyltransferase (DGAT); the ER chaperone HSPA5 (BiP/GRP78) (35); the dual specificity phosphatase DUSP1 (36); mutant variants of the cystic fibrosis transmembrane regulator CFTR (37); mutant variants of the serine protease inhibitor neuroserpin (38); prion proteins (39); mutant variants of superoxide dismutase and the deubiquitylation enzyme Ataxin-3 (40); the immune coinhibitory molecule B7-H4 (41); and gp78 itself (13, 14). This diverse substrate specificity shows that gp78 has broad influences on pathways driving metabolism, molecular signaling, proliferation, immune responses, and adaptation to cellular stress (16, 17, 28, 31, 42).

In addition to its role as an ER-associated E3 ligase, gp78 has also been linked, in past studies, to binding activity for the autocrine motility factor (AMF) (43). This association was based primarily on observations using the monoclonal antibody (3F3A), an antibody raised against lectin-isolated melanoma cell surface glycoproteins (44). Though the epitope bound by 3F3A remains unknown, this antibody was shown to stimulate melanoma cell migration in a manner similar to AMF (44, 45), and 3F3A immunoblot reactivity could be blocked by conditioned media containing AMF-like (AMFL) activity (44). Despite its undefined epitope, 3F3A immune cross-reactivity was later shown to colocalize to a smaller population of ER-associated gp78 in cells overexpressing recombinant gp78 (46, 47). Thus, it remains unclear whether 3F3A recognizes gp78 or a smaller population of AMF binding activity that associates with a discrete fraction of gp78 (46, 47).

Using an epitope-defined antibody raised against a known peptide sequence in the C-terminus of gp78 (13, 14, 24), it was later found that gp78 specifically targets the tumor suppressor protein CD82 for degradation in sarcoma cells, thus providing the first implication of a role for gp78 in human cancers (24). Subsequently, Martin et al. demonstrated that gp78 protein expression was significantly higher in the breast cancers of women of African ancestry compared with women of European ancestry (48). Notably, this activity was also associated with significant enrichment for an IFN-like immune gene expression signature, higher macrophage infiltration, and increased stromal microvascular density in patients of African compared with European ancestry (48).

In this current study, we assess the role of gp78 in breast cancer by characterizing its protein expression in a large (>500 patients) cohort of patients with breast cancer and defining its association with patient and tumor characteristics and features, including survival, grade, subtype, race/ethnicity (self-identified), and gene expression. The analysis confirms that gp78 is expressed at a higher level in the breast cancers of women of African descent, is associated with gene expression patterns that are predictive of tumor recurrence and response to therapy, and demonstrates that gp78 independently predicts poor breast cancer survival in women of African ancestry even after adjusting for age, stage, grade, and subtype.

## Results

### The E3 ligase gp78 is expressed at higher protein levels in breast cancers compared with normal breast and is associated with poor overall survival.

**Figure 1:**
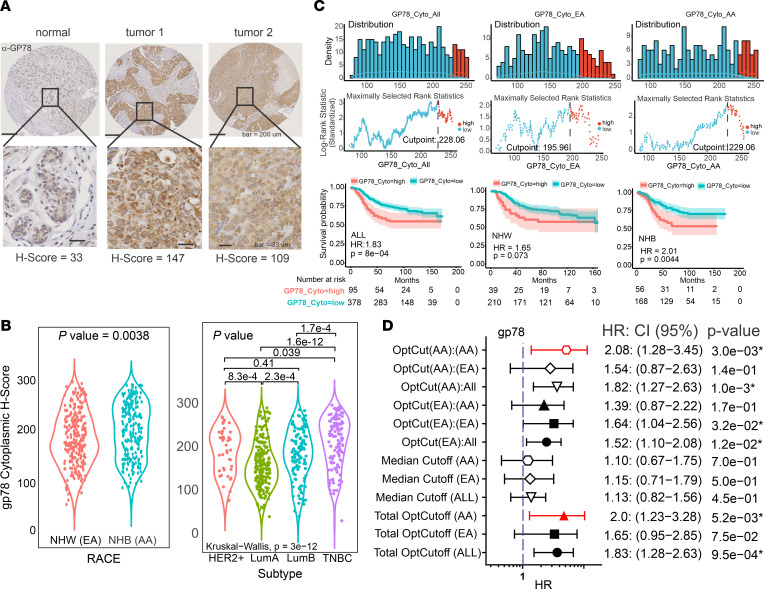
Gp78 protein is expressed at higher levels in the breast cancers of women of African ancestry and differentially predicts survival base on race. (**A**) Quantitative IHC staining of tissue microarray cores of normal versus different breast cancers. Differences in staining intensity are indicated by H-score (see text). Scale bar: 200 μm (top); 33 μm (bottom). (**B**) Left, violin plot quantitative comparison of protein expression of gp78 in the breast cancer of African American compared with European American patients. Right, violin plot comparison of relative expression of gp78 protein in different breast cancer subtypes. (**C**) Top, histogram of maximally ranked statistics analysis (6, 11) to determine optimal gp78 cutpoint. Bottom, Kaplan-Meier plot survival analysis showing the relationship between high versus low gp78 expression and survival. Middle row shows H-score versus log rank statistic. (**D**) Forest plot analysis indicating the hazard ratio, *P* value, and 95% CI for different gp78 protein cutoffs in the total cohort or the cohort separated by race using optimized or median cutoffs as indicated. Optcut(AA):(AA) represents the optimal cutoff generated only using the AA population and then applied to AA samples for survival analysis. Optcut(AA):(EA) represents the AA optimal cutoff applied to only EA samples for survival analysis. Optcut(ALL) is when the cutoff is determined from the total cohort. Red lines indicate values the showed significant survival difference.

To determine the predictive value of gp78 expression in mammary malignancies, we analyzed a previously established racially diverse breast cancer cohort of patients (*n* = 560) residing in a designated health disparities catchment area in Eastern North Carolina (median follow-up, 8.5 years) and arrayed in tissue microarray (TMA) format (6, 11, 49, 50) to define the association between gp78 protein levels and breast cancer patient tumor characteristics and survival. The expression of gp78 was measured by IHC using a quantitative digital pathology platform in which pathologist-annotated regions of tumor were scored in terms of the IHC staining level assessed by an increasing pixel intensity stratification: 0, 1^+^, 2^+^, or 3^+^ (6, 11, 49, 51). The percent of cells with these intensities were then used to assign protein expression values based on a continuous metric referred to as the H-score (0–300 scale) using the following equation: (H-score = 3 [%3^+^] + 2 [%2^+^] + 1 [%1^+^]) (6, 11, 49). Using the Aperio platform, digital scoring for gp78 was strongly correlated with the manual score provided by a pathologist (adjusted Pearson’s *R*^2^ = 0.93) (Supplemental Figure 1A; supplemental material available online with this article; https://doi.org/10.1172/jci.insight.157465DS1). Compared with normal breast, gp78 protein is expressed at substantially higher levels in breast cancer (Figure 1A). When compared by self-identified race, gp78 shows higher expression levels in women of African ancestry in contrast to European ancestry (Figure 1B, left). Notably, gp78 is more expressed in the most aggressive forms of breast cancer, characterized by rapid growth and metastatic spread, including triple-negative breast cancer (TNBC), luminal B, and the human epidermal growth factor receptor 2 (HER2) overexpressed subtypes (Figure 1B, right). Accordingly, as shown in Figure C, using gp78 H-score cutoffs determined by the methods of maximally determined rank statistics (6, 11, 52) (Figure 1C, top), Kaplan-Meier analysis revealed that high levels of gp78 protein are significantly associated with poor survival (Figure 1C, bottom). Furthermore, forest plot analysis using optimized gp78 H-score cutoff values in total patients, African American (AA) patients, or European American (EA) patients revealed that gp78 H-score cutoffs were differentially predictive of poor survival hazards based on race (Figure 1D). Specifically, cutoffs determined using the total patient cohort (total OptCutoff) were significantly predictive in the total patient cohort and African American patients, while cutoffs determined using the European American cohort (OptCut[EA]) were significantly predictive only in the total cohort and European American patients and not African Americans. Finally, cutoffs determined using the African American Cohort (OptCut[AA]) were significantly predictive of survival in the total cohort and the African American patients and not in European American patients (Figure 1D).

In the multivariate setting, after adjusting for age, BMI, RACE, menopause, subtype, hormone receptor status, lymph node status, and grade, the gp78 H-score loses statistical significance (Table 1). However, when the total patient cohort is racially stratified into European American versus African American status, gp78 protein emerges as an independent predictor of survival only in African American patients, even after adjusting for age, BMI, menopause status, and subtype (Table 1). The status of gp78 as an independent predictor of survival, based on race, remains even after additional adjustment for tumor grade (Supplemental Table 1).

**Figure 2:**
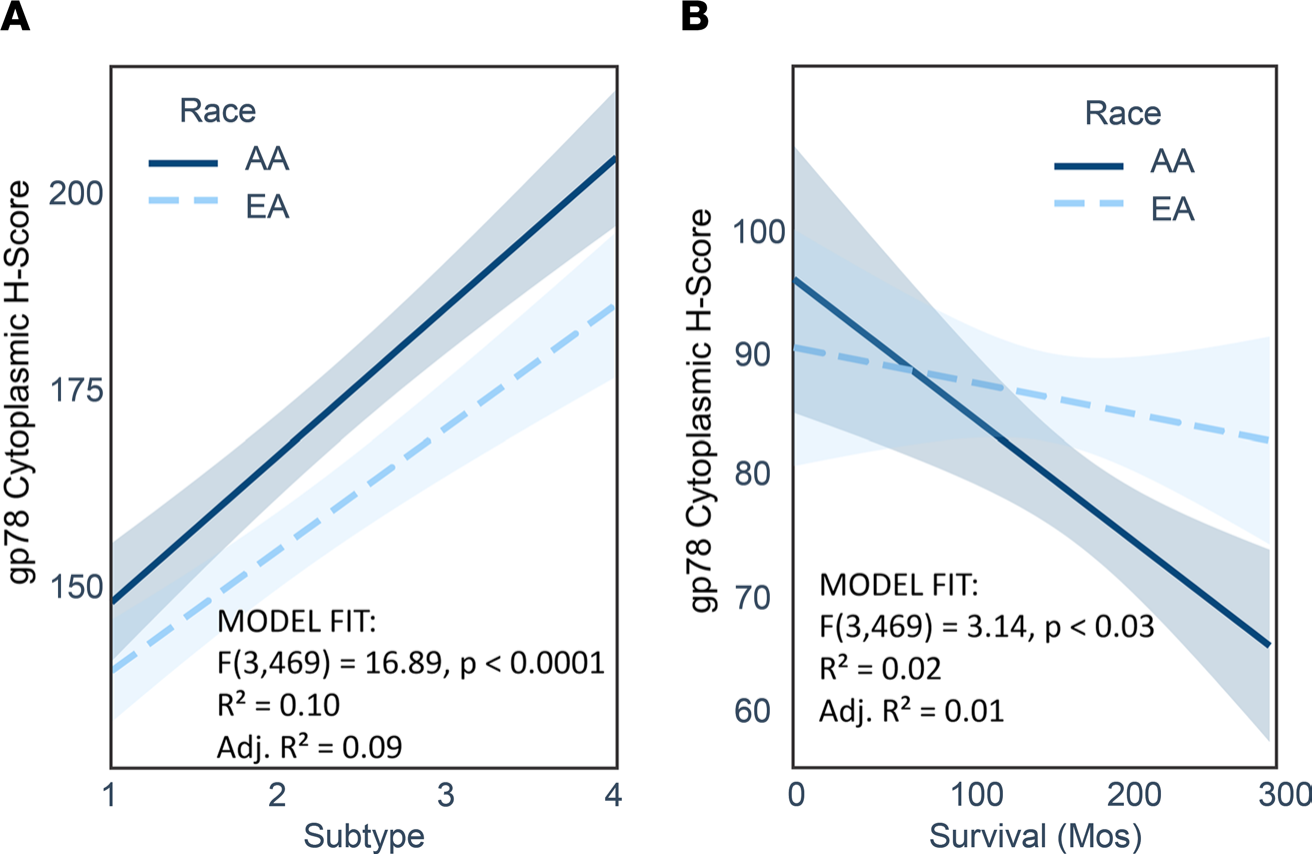
Regression profiling reveals that the relationship between gp78 protein levels and survival is influenced by race. (**A**) Interaction regression analysis showing the relationship between subtype as the independent variable (1, LumA; 2, LumB; 3, HER2^+^; and 4, TNBC) and gp78 H-score as the dependent variable in patients of African American ancestry (solid line) and patients of European American ancestry (dotted line). (**B**) Regression profiling using survival months as the independent variable and gp78 protein levels as the dependent variable in patients of African American ancestry (solid line) compared with patients of European American ancestry.

It is well known that African American women suffer a nearly 2-fold higher frequency of TNBC (3, 4, 7, 10, 53–56). Also, as demonstrated in Figure 1B, gp78 is more highly expressed in this subtype. Therefore, to rule out the possibility that the race-selective performance of gp78 is not simply explained by the disproportionate distribution of TNBC in African Americans, we introduced an interaction term to the regression model to analyze the influence of self-reported race on either the relationship between Subtype and gp78 H-score (Figure 2A) or the relationship between survival months and gp78 H-score (Figure 2B). In each case, gp78 H-score is used as the dependent variable (Figure 2 and Supplemental Figure 1, B and C). As shown by the nonintersecting regression curves (almost parallel) in Figure 2A, the relationship between subtype and gp78 protein abundance was not influenced by race. In contrast, the intersection of the regression lines shown in Figure 2B indicates that the association between survival and gp78 was modified by race. Though these trends do not rise to statistical significance (*P* value race versus subtype = 0.46; and *P* value of gp78 versus race = 0.23; Table 2), the trends reflect a difference in the influence of race on the relationship between subtype and gp78 abundance (Figure 2A) compared with influence of race on the relationship between survival and gp78 levels (Figure 2B and Supplemental Figure 1). Thus, in addition to having higher gp78 protein levels, the impact of gp78 on survival is higher in patients of African ancestry compared with European ancestry. In other words, patients of African ancestry tend to show a greater survival hazard (shorter survival) than women of European ancestry for each unit increase in tumor gp78 protein expression (Figure 2B). In contrast, stratification of patients by high versus low mRNA expression does not predict breast cancer survival and is not correlated with protein expression (Supplemental Figure 2).

### Patients stratified by gp78 protein expression show enrichment for multiple pathways associated with stress, immune responses, metabolism, cell proliferation, and intracellular signaling.

**Figure 3:**
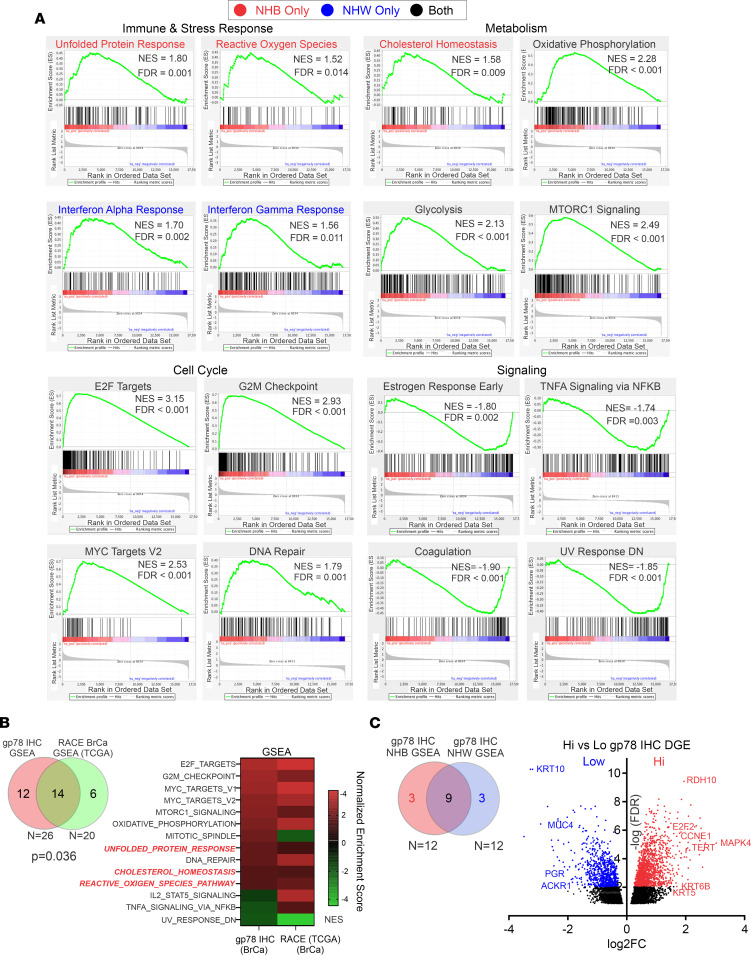
Patients with breast cancer expressing high levels of gp78 are enriched in the pathways that drive the immune, stress, metabolic, cell cycle, and cell signaling pathways. (**A**) GSEA using RNA-Seq differential expression data of patients stratified by high versus low gp78 expression (based on the median). (**B**) Overlap between gene sets enriched (FDR < 0.05) in patients stratified by gp78 expression and gene sets enriched in (FDR < 0.25) African American compared with European American patients with breast cancer. (**C**) Left, overlap between GSEA enrichment analysis (FDR < 0.05) based on gp78 protein stratification in African American (NHB) compared with European American (NHW) patients with breast cancer. Right, volcano plot of differential gene expression in patients median stratified by high versus low gp78 protein.

To define the functional state of breast cancer cells expressing high levels of gp78 protein, we leveraged the availability of RNA-Seq gene expression data for a portion of the breast cancer cohort (*n* = 147). Analysis by gene set enrichment profiling, based on the differential gene expression pattern of patients stratified by gp78 H-score using the optimal cutoff of all samples, shows enrichment for multiple gene sets representing pathways important in cellular immune responses, stress responses, metabolism, cell cycle, and cellular signaling (Figure 3A, Supplemental Figure 3, and Supplemental Table 2). Notably, several of the gene sets enriched by the stratification of gene expression according to gp78 H-score were also enriched in a recent study of TCGA data that examine gene set enrichment profiles based on the gene expression ranking of patients with breast cancer stratified by race (Figure 3B) (57). Specifically, 14 of 20 gene sets determined to be differentially enriched in breast cancer based on race were also differentially enriched in our breast cancer cohort stratified by gp78 protein expression (Figure 3B and Supplemental Figure 4). Interestingly, a partitioned comparison of the gp78-based gene set enrichment analysis (GSEA) of African American (non-Hispanic Black [NHB]) compared with European American (non-Hispanic White [NHW]) patients reveals an overlap of 9 of 12 gene sets enriched by GSEA (Figure 3C and Supplemental Table 3). Gene sets unique (FDR < 0.05) to the NHB GSEA (indicated in red in Figure 3, A and C, and Supplemental Table 3), and those unique (FDR < 0.05) to the NHW GSEA (indicated in blue in Figure 3, A and C), demonstrate that the UPR, cholesterol metabolism, and reactive oxygen species are more significantly enriched in African American patients (Figure 3, A and C, and Supplemental Table 3). In contrast, gene sets that are differentially overrepresented in European American patients include IFN-α and IFN-γ responses and heme metabolism (Figure 3, A and C, and Supplemental Table 3). Interestingly, a similar GSEA of the cohort stratified by gp78 mRNA (*RNF45*) demonstrates a comparable overlap with the TCGA breast cancer GSEA stratified by race (Supplemental Figure 5, compared with Figure 3B). However, the correlation between the *r*ace-based TCGA breast cancer GSEA and the gp78 protein-based GSEA (Pearson’s *r* = 0.5567), is higher compared with the gp78 mRNA-based GSEA (Pearson’s *r* = –0.1195) (Supplemental Figures 4 and 5). This is consistent with the lack of correlation between gp78 protein and gp78 mRNA (excluding the extremes of expression) in the patient samples (Supplemental Figure 2A). Finally, an inspection of the differential gene expression of patients stratified by gp78 protein and displayed as a volcano plot shows stratified expression of classic estrogen receptor–positive versus estrogen receptor–negative markers (Figure 3C).

### Gp78 protein expression is highly correlated with features common to more aggressive breast cancers.

**Figure 4:**
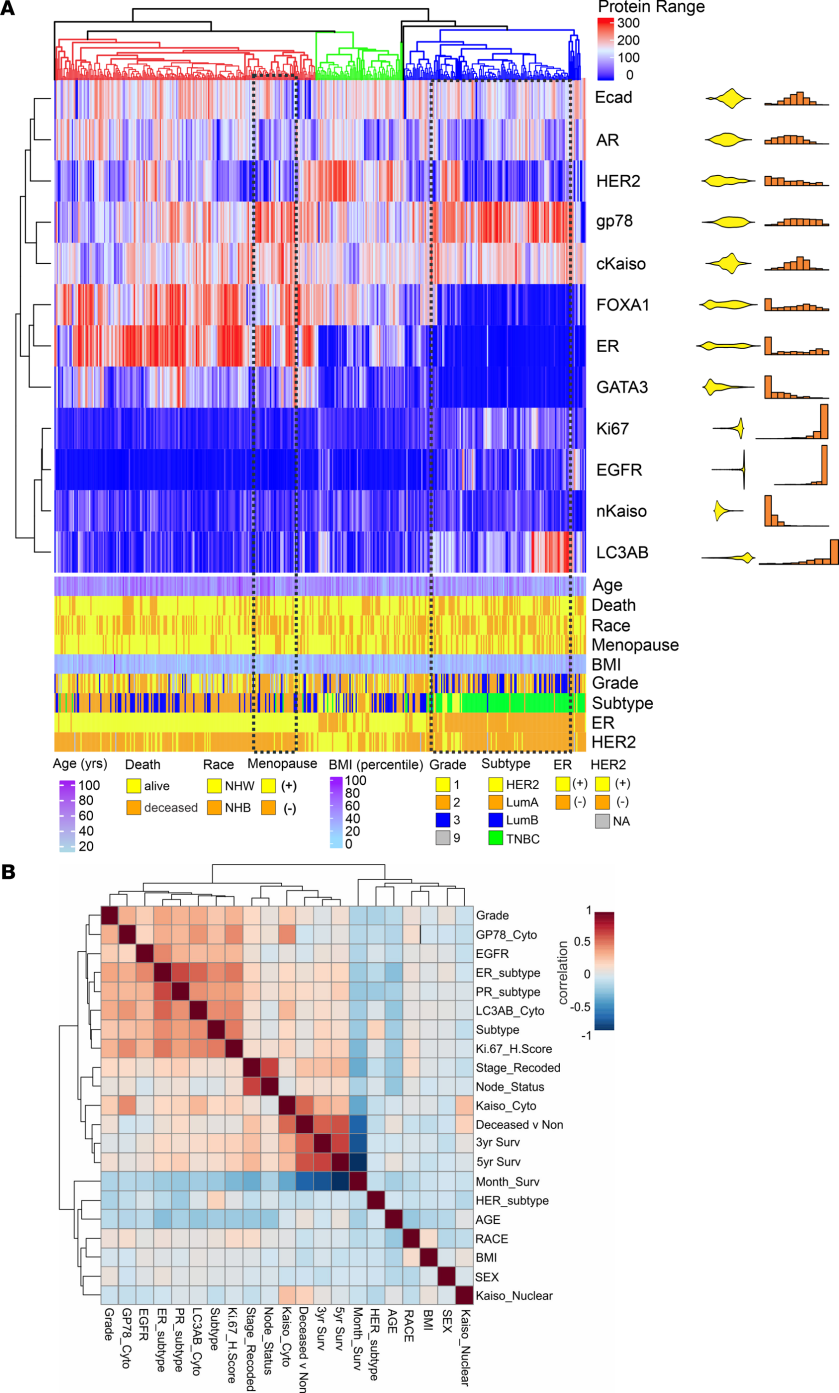
Gp78 protein is associated with multiple features commonly enriched in triple-negative breast cancer. (**A**) Heatmap of unsupervised hierarchical clustering based on breast cancer biomarker H-scores (top) with patient tumor and clinicopathological features (bottom). The H-score distribution for each biomarker is shown to the right of the heatmap with density distributions and histogram plots. (**B**) Correlation coefficient plot of patient features and tumor biomarkers arranged by hierarchical clustering.

To define both patient and tumor parameters that are associated with increased expression of gp78, we profiled tumor and patient characteristics comparing biomarkers commonly associated with more aggressive forms of breast cancer by unsupervised hierarchical clustering (Figure 4A). This analysis highlights the association of gp78 protein expression with TNBC and other markers commonly associated with TNBC or more aggressive cancers, including cytoplasmic Kaiso (cKaiso) (11), epithelial growth factor receptor 1 (EGFR1), Ki-67, and LC3A/B (11), but also demonstrates non-TNBC clusters composed of a mixture of HER2, luminal A (LumA), and LumB subtypes that express high levels of gp78 (Figure 4A). Notably many non-TNBC tumors also showing high gp78 protein are overrepresented in patients of African (NHB) ancestry (Figure 4A, highlighted by the dotted line). These associations are further summarized by the correlation coefficient plot in Figure 4B.

### Gene modules derived from patients stratified by gp78 protein expression predict recurrent disease and response to therapy.

**Figure 5:**
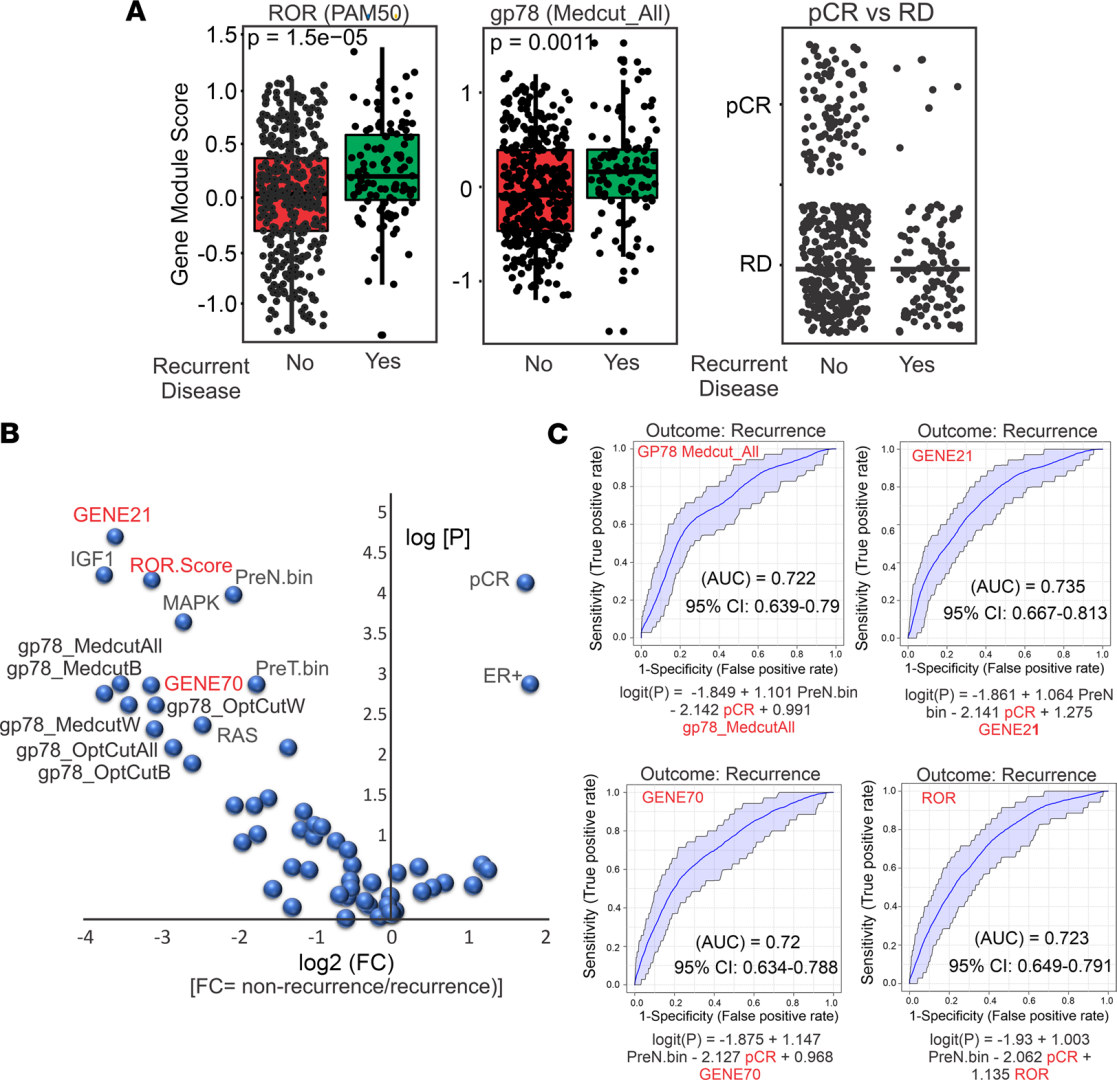
Gene modules derived from stratification by gp78 protein expression are predictive of recurrent disease. (**A**) Box plot comparing patient scores based on PAM50 (left) compared with gene modules derived from patients stratified by gp78 protein using median cutoff (center) and the pathological complete response (pCR) status of patients in patients with recurrent and nonrecurrent breast cancer. (**B**) Volcano plots of scores of patients with nonrecurrent versus a patient with recurrent (log_2_ fold change) disease with *P* values using patients scored by gp78 modules and known gene signatures. (**C**) Receiver operating curve analysis shows AUCs from logistic regression analysis using gp78 median cutoff modules, compared with Gene21, Gene70, and ROR (OncotypeDx, MammaPrint, and Prosigna) gene signatures to predict recurrence.

Using the concept of coexpression modules or lists of genes with highly correlated expression (58–60), we leveraged the available RNA-Seq data on this cohort (6, 11, 49) to construct gene modules (58) composed of genes that are strongly correlated in patient samples expressing high levels of gp78 protein (Figure 5). These gene modules were then used to perform a comparative analysis using breast cancer gene expression, treatment, and outcome data from publicly available clinical trial databases (58, 59, 61–65). Compared with the PAM50 risk-of-recurrence (ROR) score (ROR/Prosigna) (66), the gp78 gene modules derived from the differential gene expression pattern of patients with breast cancer, stratified using the median cutoff for gp78 expression in the total cohort (labeled as Medcut_All), shows significantly higher scoring in patients with recurrent disease. As expected, in the neoadjuvant setting, those patients with pathological complete response (pCR) show dramatically lower recurrent disease (Figure 5A, right). Notably, the gp78 gene module score stratifies or predicts those patients with nonrecurrence versus recurrence of breast cancer using a variety of gp78 H-score cutoffs (Figure 5B). Furthermore, receiver operating curve (ROC) analysis for the gp78 gene module yields AUC values predicting disease recurrence comparable with those signatures currently in clinical use, including the Prosigna PAM50 ROR score (66), the Oncotype GENE21 score (67), and the GENE70 MammaPrint score (68) (Figure 5, B and C, and Supplemental Figures 6–8).

**Figure 6:**
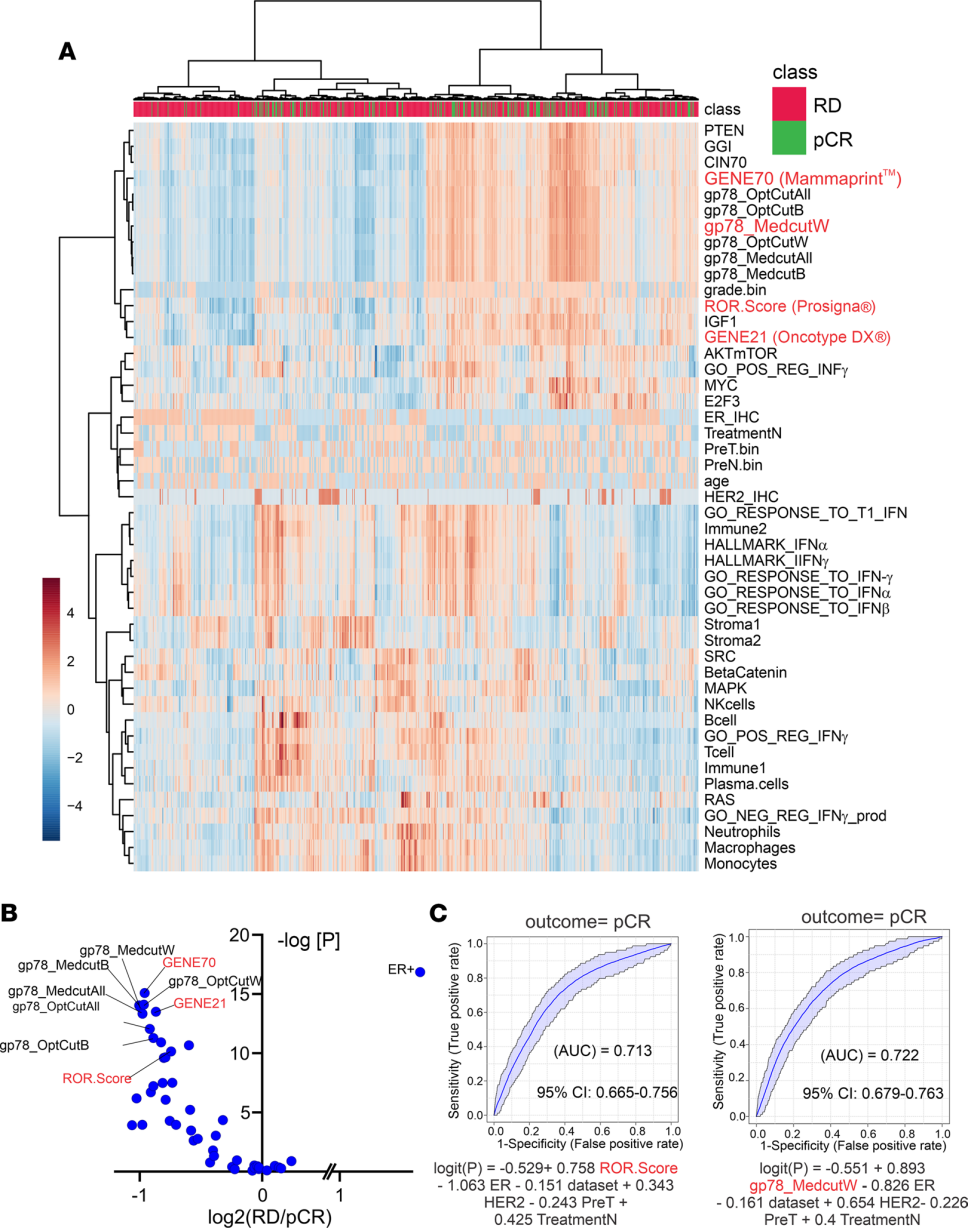
Gp78-derived gene modules predict pCR and cluster closely with established predictive gene signatures. (**A**) Hierarchical clustering of patient scores based on gp78 modules and other predictive modules. (**B**) Volcano plot of patient gp78 gene modules scores and other gene signature scores in patients with residual versus pCR (log_2_ residual disease/pCR). (**C**) Receiver operating curve comparison of regression analysis prediction of pCR using ROR (Prosigna) versus gp78 modules (MedcutW). See also Supplemental Figure 8.

Hierarchical clustering analysis, comparing patient gp78 gene module scores with other previously described gene signatures (58–60), demonstrates that gp78 scores are more closely correlated with the gene expression scores generated by the Gene70 (MammaPrint) (68), the CIN70 (chromosomal instability) (69), genomic grade index (GGI) (70), and PTEN (71) gene tests (Figure 6A and Supplemental Figures 7 and 8). Notably, patient scores highly correlated within the gp78-derived module clusters show higher frequencies of pCR. Moreover, the ability of the gp78 modules to predict that pCR is comparable both in magnitude and significance to the 3 gene panels (Prosigna, OncotypeDX, and MammaPrint) currently in clinical use (Figure 6, B and C, and Supplemental Figure 8) (66, 67, 72).

### Elevated levels of tumor gp78 are correlated with specific immune spatial, morphological, and gene expression attributes in the tumor microenvironment.

**Figure 7:**
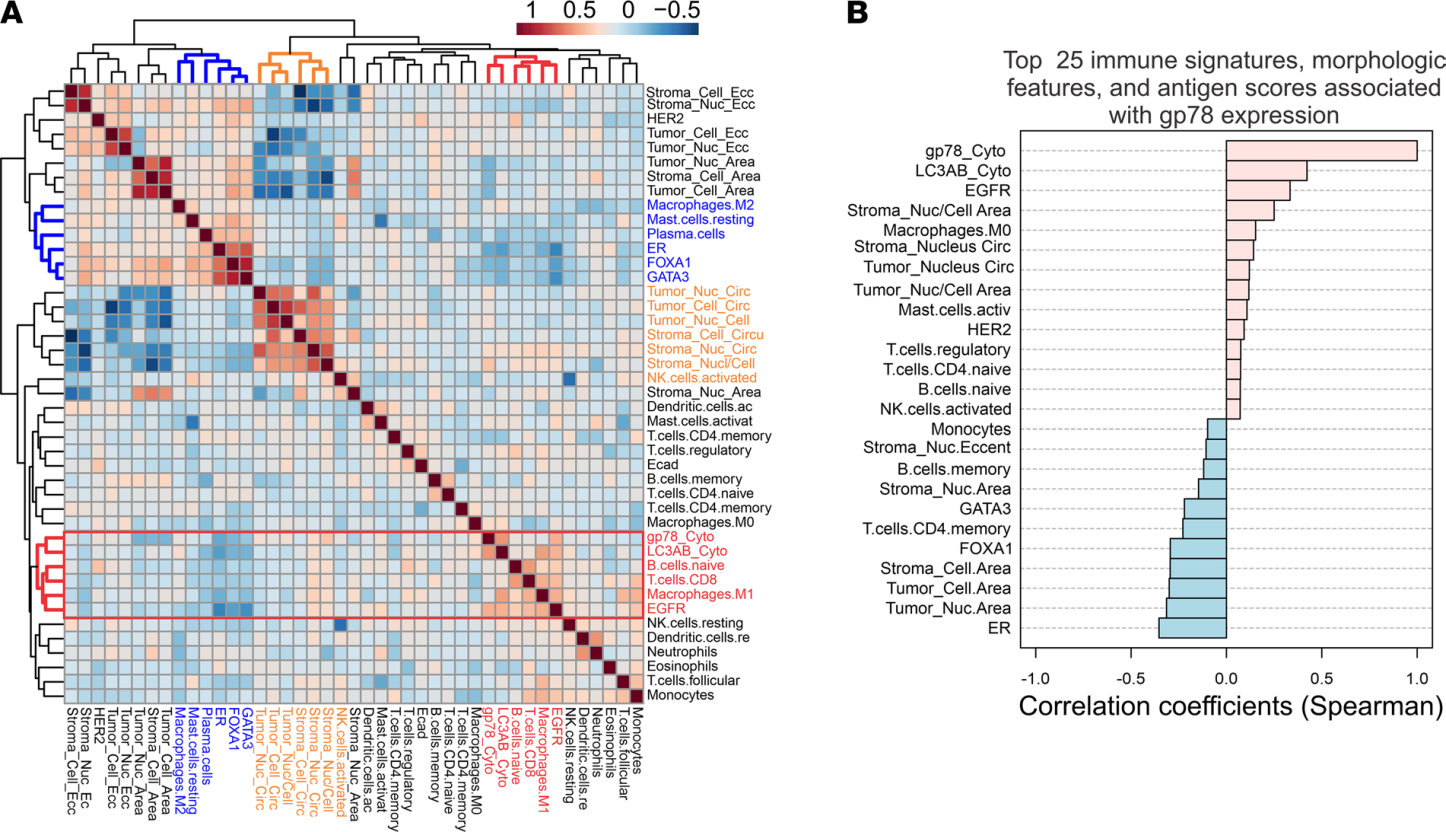
Elevated levels of tumor gp78 are correlated with specific morphological and gene expression attributes in the tumor microenvironment. (**A**) Hierarchical clustering of correlation of biomarker values, tumor, and stromal morphological features, as well as deconvolved immune expression (CIBERSORT) signatures and gp78 protein scores. (**B**) Correlation of the top 25 immune signatures, morphological features, and antigen scores with gp78.

**Figure 8:**
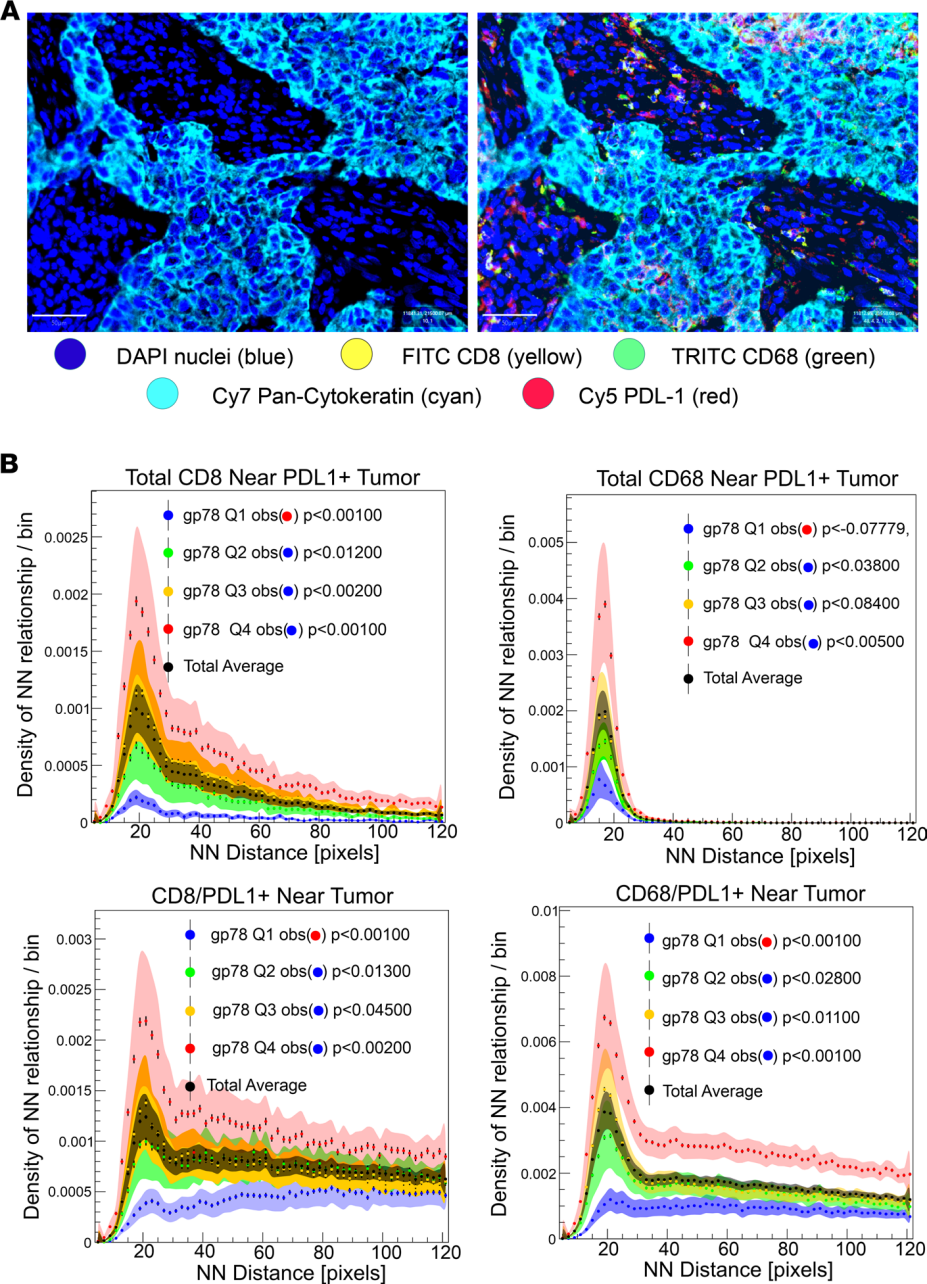
Spatial analysis of the association of gp78 protein expression with proximities of PD-L1–positive CD8, CD68, and tumor cells within the TME. (**A**) Multiplex immunofluorescent image of patient tumors stained for CD8, CD68, Pan-cytokeratin, and PD-L1. (**B**) Distance distribution profiles of proximity frequencies between CD8, CD68, and tumor cells based on their PD-L1 expression. Distances are represented as pixels (11). Scale bar: 50 μm.

Previous studies have shown that breast cancers, arising in women of African ancestry, are associated with specific stromal and immune responsive features in the tumor microenvironment (7, 48, 73–75). African American women tend to have higher levels of protumorigenic M2 macrophages, Tregs, microvasculature, and circulating proinflammatory cytokines (7, 48, 73–75). To evaluate the association between gp78 and multiple features within the tumor microenvironment, we used a multiomic approach that combined: (a) immune features of the tumor microenvironment inferred from available RNA-Seq data to estimate the relative abundance of different immune cell types using CIBERSORT as previously described (7, 76); (b) quantitative morphological assessment of the stromal and epithelial nuclear and cytoplasmic features (77, 78); and (c) IHC-based protein biomarker profiling (6, 11, 49, 50) (Figure 7). Integration of these values followed by normalization and unsupervised hierarchical clustering (Figure 7A) and correlation analysis (Figure 7B) reveals associations linking gp78 with LC3A/B, a naive B cell gene signature, a CD8 T cell gene signature, M1 macrophages, EGFR protein abundance, and a higher stromal infiltration with cells containing a high nuclear/cytoplasmic ratio and nuclear circularity consistent with lymphoid infiltration. This observation is consistent with Figure 4 and the recently published observation that LC3A/B is highly associated with estrogen receptor–negative tumors and TNBC (11). Moreover, high LC3A/B expression has been associated with features of an immunosuppressed tumor microenvironment characterized by increased frequencies of PD-L1^+^ CD8 T cells and CD68 macrophages in close spatial proximity with tumor, although there was nonsignificant proximity of CD8 and CD68 cells with PD-L1^+^ tumor (11). Interestingly, a similar proximity-based analysis of these linkages (Figure 8) shows that high gp78 is associated with both increased proximity of PD-L1^+^ CD8 and CD68 cells to tumor, as well as increased proximity of CD8 and CD68 cells near PD-L1^+^ tumor, suggesting an increased likelihood of immune suppression (Figure 8B). In summary, gp78 expression is associated with activation of both innate and adaptive immunity pathways in the breast cancer microenvironment but also demonstrates significant features suggesting immune suppression.

## Discussion

This study is the first to our knowledge to show a statistically significant correlation between breast cancer gp78 protein expression and patient survival using an epitope-defined antibody against the E3 ligase, gp78. We begin by providing evidence that gp78 was significantly upregulated in breast cancers, compared with normal breast; we then revealed that gp78 expression was also significantly more elevated in the tumors of African American women as opposed to those of European ancestry. We then demonstrated that gp78 expression was higher in more aggressive breast cancer subtypes and predicted poor breast cancer survival (Figure 1). Notably, in the multivariate setting, after adjusting for age, grade, and subtype, we demonstrated that gp78 was an independent predictor of poor survival specifically in women of African ancestry (Table 1). GSEA using the RNA-Seq data of patients with breast cancer stratified by high versus low gp78 protein demonstrates that tumors expressing high levels of gp78 protein showed much greater activation of immunorelated, metabolic, cell-cycle, and other cell-stress adaptive pathways including the UPR and cholesterol homeostasis (Figure 3 and Supplemental Figure 3). These pathways are consistent with both the substrate specificity of gp78 and the genome-wide association studies–defined (GWAS-defined) linkages between gp78 and single nucleotide polymorphisms (SNPs) — rs150646468, rs2432539, rs112233856, rs2587865, and rs111283203 — associated with IFN-γ measurements, breast carcinoma, apolipoprotein A1 levels, triacylglycerol levels, and type II diabetes mellitus, respectively (see GWAS catalog https://www.ebi.ac.uk). Furthermore, greater than 70% of the gene sets, enriched by gp78 protein expression, are also enriched in patients with breast cancer, stratified by race, described in a previous study using TCGA data (57) (see Figure 3 and Supplemental Figures 4 and 5). The correlation between gp78 expression and other predictive protein biomarkers, by unsupervised hierarchical clustering, reveals a strong association with characteristics linked to TNBC, including, EGFR, Ki-67, and the more recently described extracellular vesicle targeting and loading protein LC3A/B (11, 79) (Figure 4). Gp78 gene modules assembled from the correlated gene expression patterns of patients stratified by gp78 protein are highly predictive of recurrence and response to standard-of-care therapy (pCR) at a level that is comparable with established gene signatures such as Prosigna, OncotypeDx, and MammaPrint (Figures 5 and 6). Finally, examination of the tumor microenvironment shows a high correlation with activation of innate immune responses revealed by the increased lymphoid and macrophage (M1) infiltration seen in the tumors of patients with high levels of gp78 protein (Figure 7A and Figure 8B).

It is notable that features of immune suppression, characterized by increased proximities of CD8 and CD68 cells to PD-L1–expressing tumor and the increased infiltration of PD-L1^+^ macrophages and CD8 cells, are observed in patients expressing high levels of gp78 (Figure 8B). These findings suggest gp78 may exert immune suppressive influence on the tumor microenvironment. Such observations are consistent with the association between gp78 and LC3A/B levels, whose expression has recently been shown to be associated with an immunosuppressed tumor microenvironment characterized by increased PD-L1 tumor expression and infiltration with PD-L1^+^ CD8 cells (11, 80), as well as the recent observation that gp78 is linked to increased tumor PD-L1 expression through its degradation of B7-H4 (41). Although B7-H4 has been suggested to be an immune checkpoint protein, several studies have shown an inverse relationship between B7-H4 expression and PD-L1 (81–83). This explains the paradoxical relationship between gp78-dependent degradation of B7-H4 and the tumor microenvironment, and it is consistent with observations that decreased levels of tumor B7-H4 are associated with increased stromal lymphocyte infiltration (41, 81–83). Thus, gp78-expressing tumors are not classically immunologically “cold” but instead represent a combination of features of immunologically “hot” tumors with those of immune suppression — an attribute often associated with tumors with a high mutational burden (80). This trend is demonstrated by the lack of a typical immune-exhausted phenotype characterized by increased PD-1^+^ memory T cells, in close proximity to tumor, seen in patients expressing high levels of gp78 (Supplemental Figure 9). The higher tumor mutational burden often seen in TNBC would be expected to increase ER stress, the resulting UPR, and levels of tumor-infiltrating lymphocytes (9). Given these correlations and the known role played by gp78 in activation of the STING response (30), it is likely that gp78 tumor will show a high correlation with other signatures linking the DNA damage response to immune reactivity — e.g., the DNA damage immune response (DDIR) (84–86). This presumed association is consistent with the high correlation between the gp78 modules and gene expression signature/modules linked to DNA damage including CIN70 (chromosomal instability) (69) and GGI (70) (Figure 6 and Supplemental Figure 9). Finally, the substrate specificity of gp78 for CYP3A4 may also have broad ramifications since the cytochrome P450 is the dominant enzyme responsible for the metabolism of over 50% of clinically relevant drugs (87). This property is likely to influence treatment response in a variety of therapeutic settings.

The process through which gp78 protein levels are regulated is complex. Gp78 is autoubiquitinated and a substrate for other E3 ligases associated with ERAD (13, 14, 88), and its stability is induced by ER stress (89). Recently, gp78 has also been shown to be stabilized by the ubiquitin peptidase USP34 (89). The observation that USP34 is induced under conditions of DNA damage, where it associates with and stabilizes RNF168 (90), provides a plausible link whereby gp78 levels may also be induced in response to DNA damage. This regulatory linkage would serve to increase gp78 levels in anticipation of the upregulated ER stress and UPR that would be induced by gene mutation. Such a connection is consistent with the association between gp78, TNBC, and gene signatures for chromosome instability and GGI described above.

Although gp78 has been previously associated with AMF binding activity (the gp78 Human Genome Organization Gene Nomenclature Committee–approved [HNGC-approved] gene name is AMF receptor [AMFR]), this conclusion is based on an antibody (3F3A) that recognizes an unknown epitope (44). Direct comparison of 3F3A reactivity with antibodies raised against known gp78 epitopes, by both IHC and Western blotting, shows little correlation with the antibody used in this current study, those that have been previously published (13, 14, 24, 48), and other gp78 epitope–defined antibodies that are commercially available (Supplemental Figures 10–12). It will be important that future studies examining the role of gp78 in human malignancies rely on data generated from similar epitope-define antibodies (91).

Finally, the difference in the predictive value of gp78 based on race is compelling, and there remains a possibility that gp78 protein scores or gene modules derived from gp78 may be differentially predictive of recurrence and response to therapy based on race. Testing this hypothesis will require further validation of this observation in other breast cancer cohorts with treatment, survival, and therapeutic response data provided by clinical trials with more diverse patient enrollment and access to tissue. Future efforts to examine the association between gp78 expression and metabolic features of the tumor and tumor microenvironment using recent advances in metabolomics analysis will be a priority. The observation that gp78 protein remains the sole independent predictor of survival in women of African ancestry even after normalizing for TNBC subtype and grade (Table 1 and Supplemental Table 1) suggests that gp78^hi^ tumors may have arisen in early ancestral African populations as a feature with universally associated aggressive breast cancers regardless of subtype prior to the evolution of TNBC, where gp78^hi^ tumors may have been a more predominant subtype prior to the out-of-African events believed to occur in the late Pleistocene epoch (92, 93). This is consistent with the lost significance of the TNBC subtype as an independent predictor in women of African ancestry after normalizing for gp78 protein expression (Table 1). Similarly, correlation plots in which percent African, Asian, European, South Asian, and admixed Native American genetic ancestry is quantitatively correlated with gp78 protein levels, and self-identified race provide support for this idea (Supplemental Figure 13). Future investigative GWAS aimed at defining how ancestry-dependent genetic loci, SNPs, and expression quantitative trait loci related to different cellular and molecular properties that promote elevations in gp78 protein will shed more light on this hypothesis.

## Methods

### Study population, tissue microarray construction, and analysis.

Following IRB approval from East Carolina University and the NIH Intramural Research Program, deidentified formalin-fixed and paraffin-embedded (FFPE) tissue samples and deidentified clinical information abstracted from the medical records were requisitioned and initially procured for patients with breast cancer who underwent surgery for Stage 0 to Stage IV breast cancer between 2001 and 2010 at Pitt County Memorial Hospital (now Vidant Medical Center) in Greenville, North Carolina, USA. Race, ethnicity, or ancestry was self-reported at the initial visit and captured in the medical record. Survival was recorded retrospectively from the medical records and the cancer registry. The median follow-up was 8.5 years. Tissue microarrays were constructed using 1 mm cores as previously described (94, 95), with a complete representation of 555 patients.

### Methods for IHC.

Breast tumor tissue microarrays were stained, using monoclonal primary antibodies, ERα at 1:35, low pH (catalog MA5- 13191, DAKO); EGFR at 1:500, high pH (catalog M7298, DAKO); E-Cadherin at 1:50, high pH (catalog M361201-2, DAKO); Ki-67 ready to use, at low pH (MIB-1, Dako); and GATA3 at 1:50, high pH (catalog sc-268, Santa Cruz Biotechnology Inc.). Also, polyclonal primary antibodies were used, FOXA1 at 1:10,000, high pH (catalog ab23738, Abcam) and the ready-to-use antibody c-erbB–oncoprotein at a high pH (catalog A0485, DAKO). The gp78 antibodies were generated and affinity-purified from rabbits injected with a peptide containing aa 574–597 derived from the C-terminus of gp78, used at a final concentration of 0.4 μg/mL, high pH; LC3A/B antibodies (cross-reacting with both LC3 A and B isoforms) used at a final dilution of 1:3000, high pH (catalog 12741s, Cell Signaling Technology); antibodies to androgen receptor (Atlas, HPA034966) used at 1:1000, high pH; and Kaiso antibodies used at 1:1000 dilution, high pH, as previously described (11).

### Methods for clinical variables.

Median follow-up and median survival for patients were 8.5 and 6.67 years, respectively. Clinical subtypes 2 or 3 were categorized as HER2^+^ when available information in the patient medical record showed a score greater than 2 by in situ hybridization or 3+ by IHC. ER^+^ patients were classified from the medical record and confirmed by IHC; missing data were replaced by TMA

All digital scoring was performed using the nuclear and membrane algorithms provided by the Leica Biosystems Aperio software. Areas of interest were outlined by the pathologist and then scored independently by the pathologist and the respective algorithms.

### Multispectral fluorescence imaging and nearest-neighbor analysis.

We use the Ultivue UltiMapper I/O PD-L1 assay and the PD-1 assay to collect the qmIF data. This PD-L1 kit uses the following antigens: CD8, CD68, PD-L1, pan-cytokeratin (panCK), and DAPI (DNA marker). The PD-1 immune exhaustion kit uses a 4-plex panel for CD3, CD45RO, PD-1, panCK, and DAPI. The raw image data were collected at 20×. The fluorescent dye intensities are normalized to 0–255. Image analysis was performed using a commercial software package (HALO, Indica Labs) at full magnification. Point cloud generation and nearest-neighbor analysis were conducted as previously described (11).

### Biostatistical methods.

Patient baseline characteristics and disease factors were summarized using descriptive statistics. Categorical variables were compared using the 2-sided Pearson χ^2^ test. Comparison of IHC scoring was performed by a 2-tailed *t* test and plotted as previously described (96). Univariate and multivariate Cox proportional-hazards model was used to test the independent and combined prognostic values of proteins of interest with/without the presence of selected clinical variables. Spearman’s rank correlations were used to assess the relationship between protein H-score and gene expression (reads per kilobase of transcript, per million mapped reads [RPKM]) values (97). The significance of individual hazard ratios was estimated by Wald’s test. Optimal cutoff points for H-score were determined as previously described (6, 52). The solid lines and histogram present data for samples with higher (red) or lower (blue) H-scores; the dashed lines present data for samples divided into 2 groups based on the “optimal cutoff” algorithm (52). Unsupervised hierarchical clustering of IHC protein score from all breast cancer samples was performed using complete linkage and distance correlations with the number of bootstrap replications (*n* = 1000) using the pvclust R package (98). The estimated clustering stability was measured by AU (approximately unbiased) *P* value and BP (bootstrap probability) value for each cluster in a dendrogram (98).

### RNA-Seq data and analysis, nearest-neighbor analysis, genetic admixture, and GSEA.

RNA-Seq data and analysis were conducted as previously described (6, 11, 49), genetic admixture analysis (11), and GSEA were conducted as previously described (11). Briefly, for GSEA the median cutoff of protein data was used to classify patients into 2 groups based on H-scores (e.g., low versus high gp78) with defined gp78 cutoffs for H-score or mRNA abundance (RNA-Seq). A 2-tailed *t* test was performed, and all available genes were ranked according to *p* value (lowest to highest). The *P* value–ranked gene list was used for functional correlation using preranked GSEA software (http://software.broadinstitute.org/gsea/index.jsp). Genetic admixture analysis was conduct as previously described (11). Sequences were submitted to BioProject Dbase under BioProject ID PRJNA486351 and submission ID SUB4408142 for public availability.

### Statistics.

A Spearman’s rank correlation test was performed to test the relation between its protein H-score and gene expression (RPKM) values (97). A completely unsupervised hierarchical clustering approach was performed on the 486 patient sample H-scores containing complete clinical information. Complete linkage and distance correlations were used for clustering protein data with bootstrap resampling techniques. The stability of the clustering was estimated with the pvclust R package (98) available on CRAN (https://cran.r-project.org/web/packages/pvclust/pvclust.pdf). A 2-tailed *t* test was employed to test the null hypothesis (H_0_) assumption of equality of the protein values in 2 defined groups of data and is demonstrated by violin plots using R software and ggplot2 package (96).

To classify the patients into low- versus high-risk categories using selected protein H-scores, the optimal cutoff approach (52) has been used to compute optimal cutoff points for diagnostic markers with continuous values for the entire population. The same cutoff points were applied to subclasses of data — i.e., the NHB and NHW populations. In addition, we performed a prognostic value comparative analysis using optimum cutoff point based on a specific population, as well as the median of the entire population. The prognostic value of proteins or genes was calculated by univariate Cox regression. A multivariate Cox proportional-hazards model (99) was used to test the independent and combined prognostic values of proteins of interest with/without the presence of selected clinical variables. Cox models were stratified by race to account for the possible heterogeneity in patient selection or other potential confounders. The “survival” R package was used, which is available on CRAN (https://cran.r-project.org/web/packages/survival/survival.pdf). The significance of individual hazard ratios was estimated by Wald’s test.

### Study approval.

Use of human tissue in this study was reviewed and approved by the IRBs of the Intramural Research Program of the NIH, East Carolina University, and Columbia University Irving Medical Center.

## Author contributions

SKS, YCT, AMW, NV, and KG formulated and designed the study; SKS, JM, TY, and SB reviewed performed biostatistical analysis; SKS, KG, RY, and JSB wrote the manuscript; JSB, AC, SGH, JP, and JW performed staining and scoring; EJPS, AMN, ADS, CY, MBD, and VM provided scientific input; JM review statistical analysis; SMH provided reagents and tissue blocks; and MY generated and validated antibodies.

## Supplementary Material

Supplemental data

## Figures and Tables

**Table 1:**
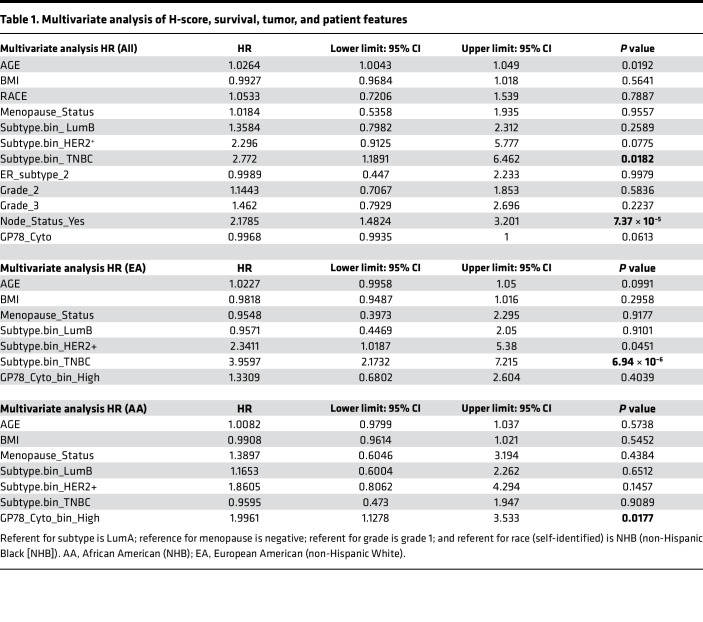
Multivariate analysis of H-score, survival, tumor, and patient features

**Table 2:**
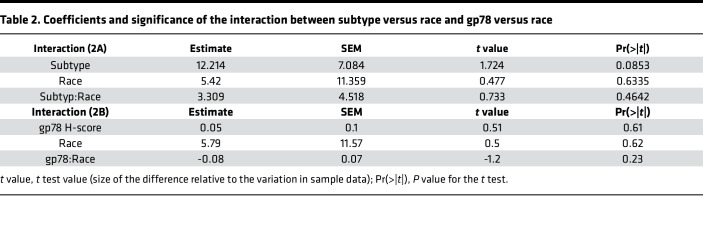
Coefficients and significance of the interaction between subtype versus race and gp78 versus race

## Version 1 05/31/2022

In-Press Preview

## Version 2 07/08/2022

Electronic publication
