# Supplementary material for: Protein expression of the gp78 E3 ligase predicts poor breast cancer outcome based on race
Source: JCI Insight. 2022 Jul 8;7(13):e157465. doi: 10.1172/jci.insight.157465 (PMC9310521; doi:10.1172/jci.insight.157465)
Supplement: Supplemental data [file jciinsight-7-157465-s047.pdf]

A

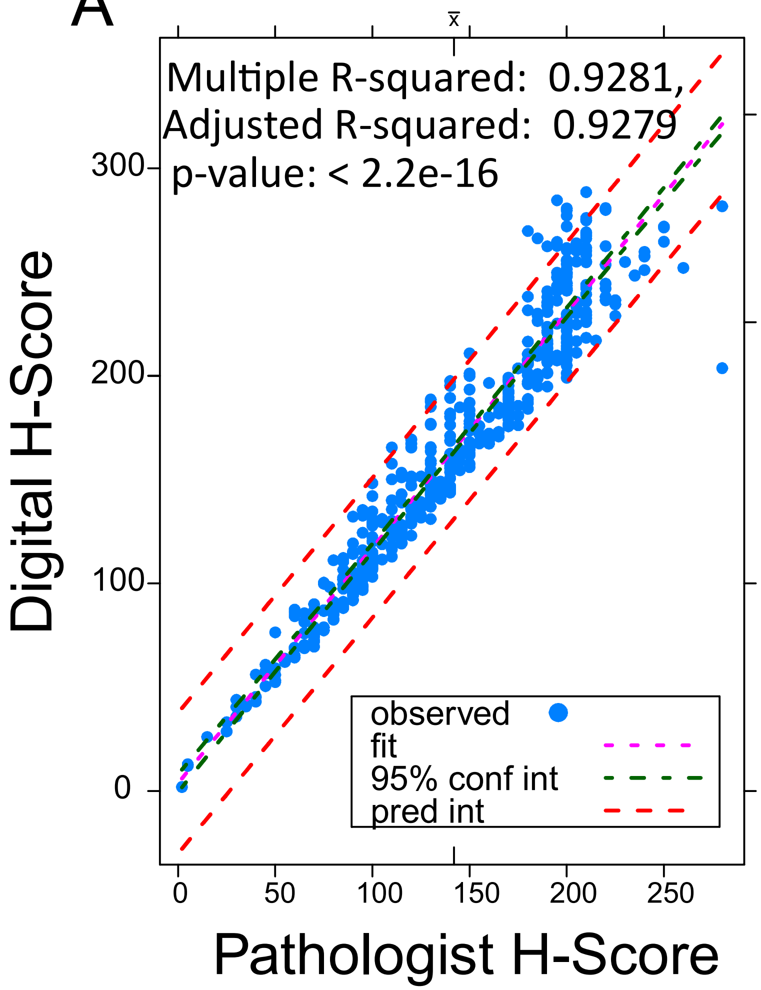

B

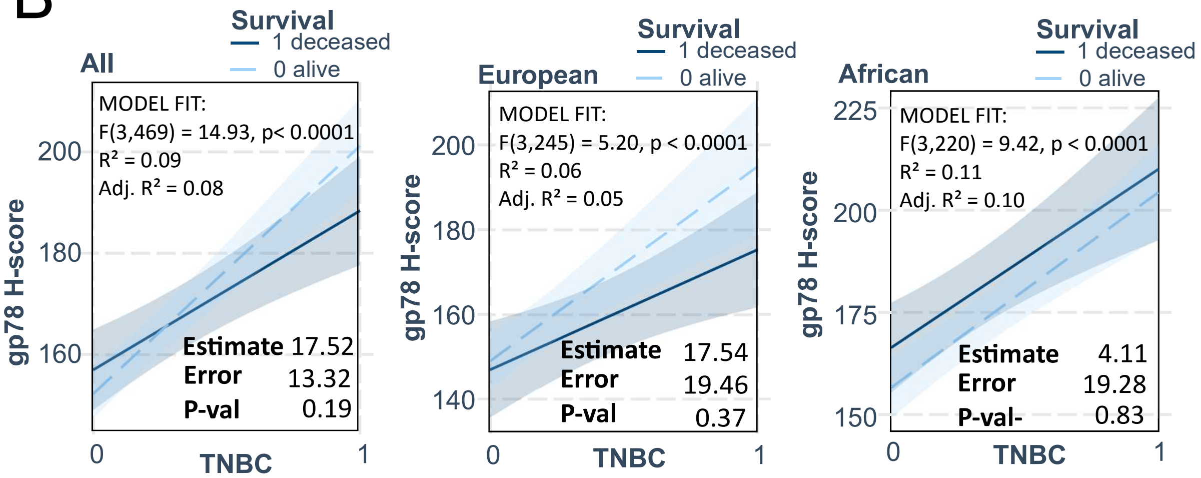

C

ALL

Interaction	Estimate	Std. Error	t-value	Pr(>|t|)
TNBC	48.97	8.29	5.91	<0.0001
Survival	4.73	7.29	0.65	0.52
TNBC: Survival	-17.52	13.32	-1.31	0.19

European

Interaction	Estimate	Std. Error	t-value	Pr(>|t|)
TNBC	45.87	13.67	3.35	<0.0001
Survival	-2.04	10.22	-0.2	0.84
TNBC: Survival	-17.54	19.46	-0.9	0.37

African

Interaction	Estimate	Std. Error	t-value	Pr(>|t|)
TNBC	48.97	8.29	5.91	<0.0001
Survival	9.78	10.38	0.94	0.35
TNBC:Survival	-4.11	19.28	-0.21	0.83

Supplementary Figure 1: Correlation between gp78 digital score and pathologists score and interaction between gp78 digital score and TNBC subtype. (**A**) Correlation is shown between digital and pathologists scores using both multiple and adjusted correlations with p-value and 95% confidence interval provided. (**B**) (right) Interaction curves showing that the relationship between gp78, TNBC, and survival in the total cohort (ALL) compared to women of European versus African ancestry demonstrating a greater interaction between TNBC and survival in European patients compared to patients of African ancestry. (**C**) Table showing that TNBC subtype is influenced by survival in the total cohort and women of European descent, but much less so in women of African ancestry.

Multivariate analysis: HR (All)	HR	CI (95%) lower	CI (95%) upper	p-value
AGE	1.0264	1.0043	1.049	**0.0192**
BMI	0.9927	0.9684	1.018	0.5641
RACE_(AA)	1.0533	0.7206	1.539	0.7887
Menopause_Status	1.0184	0.5358	1.935	0.9557
Subtype.bin_ LumB	1.3584	0.7982	2.312	0.2589
Subtype.bin_HER2+	2.296	0.9125	5.777	0.0775
Subtype.bin_ TNBC	2.772	1.1891	6.462	**0.0182**
ER_subtype_	0.9989	0.447	2.233	0.9979
Grade_2	1.1443	0.7067	1.853	0.5836
Grade_3	1.462	0.7929	2.696	0.2237
Node_Status_Yes	2.1785	1.4824	3.201	**7.37E-05**
gp78_Cyto_(High)	0.9968	0.9935	1	**0.0613**

Multivariate analysis: HR (EA)	HR	CI (95%) lower	CI (95%) upper	p-value
AGE	1.0227	0.9958	1.05	0.0991
BMI	0.9818	0.9487	1.016	0.2958
Menopause_Status	0.9548	0.3973	2.295	0.9177
Subtype.bin_LumB	0.9571	0.4469	2.05	0.9101
Subtype.bin_HER2+	2.3411	1.0187	5.38	**0.0451**
Subtype.bin_TNBC	3.9597	2.1732	7.215	**6.94E-06**
gp78_Cyto_(High)	1.3309	0.6802	2.604	0.4039

Multivariate analysis: HR (AA)	HR	CI (95%) lower	CI (95%) upper	p-value
AGE	1.0082	0.9799	1.037	0.5738
BMI	0.9908	0.9614	1.021	0.5452
Menopause_Status	1.3897	0.6046	3.194	0.4384
Subtype.bin_LumB	1.1653	0.6004	2.262	0.6512
Subtype.bin_HER2+	1.8605	0.8062	4.294	**0.1457**
Subtype.bin_TNBC	0.9595	0.473	1.947	**0.9089**
gp78_Cyto_(High)	1.9961	1.1278	3.533	**0.0177**

Multivariate analysis: HR (EA)	HR	CI (95%) lower	CI (95%) upper	p-value
AGE	1.0248	0.9974	1.053	0.0762
BMI	0.9832	0.9508	1.017	0.3214
Menopause_Status	0.8929	0.3674	2.17	0.8025
Subtype.bin_LumB	0.9738	0.4465	2.124	0.9467
Subtype.bin_HER2+	2.1747	0.8878	5.327	0.0892
Subtype.bin_TNBC	3.9022	2.0375	7.473	**4.01E-05**
Grade_2	0.8202	0.4444	1.514	0.5262
Grade_3	1.166	0.5348	2.542	0.6993
gp78_Cyto (High)	1.2385	0.6193	2.477	0.5453

Multivariate analysis: HR (AA)	HR	CI (95%) lower	CI (95%) upper	p-value
AGE	1.0053	0.9768	1.035	0.7201
BMI	0.9931	0.9633	1.024	0.6587
Menopause_Status	1.5408	0.6628	3.582	0.3152
Subtype.bin_LumB	1.0842	0.5554	2.117	0.8127
Subtype.bin_HER2+	1.4165	0.586	3.424	0.4394
Subtype.bin_TNBC	0.8016	0.378	1.7	0.5642
Grade_2	1.986	0.9562	4.125	0.0658
Grade_3	2.0083	0.8046	5.013	0.1352
gp78_Cyto (High)	1.8288	1.0267	3.258	**0.0404**

Supplemental Table 1: **(left, top)** Table showing multivariate analysis of the independent predictive value of gp78 protein levels for survival after adjusting for age, BMI, menopause status, subtype, ER status, grade, and lymph node status. **(left, middle).** Shown is the multivariate analysis of the association between gp78 and survival adjusted for age, BMI, menopause status, and subtype in women of European ancestry compared to **(left, bottom)** women of African ancestry. **(Right)** Multivariate analysis of the relationship between gp78 protein expression and survival after adjusting for age, BMI, menopause status, subtype, and grade comparing patients of European **(right, top)** versus African ancestry **(right, bottom).**

Negatively regulated					
**NAME**	SIZE	ES	NES	NOM p-val	FDR q-val
HALLMARK_EPITHELIAL_MESENCHYMAL_TRANSITION	187	-0.5422555	-2.4593184	0	0
HALLMARK_ANGIOGENESIS	32	-0.6005164	-1.9944273	0	0
HALLMARK_COAGULATION	100	-0.4565779	-1.9019318	0	9.37E-04
**HALLMARK_UV_RESPONSE_DN**	141	-0.4224794	-1.8528675	0	0.0018427
HALLMARK_ESTROGEN_RESPONSE_EARLY	192	-0.3925208	-1.8076392	0	0.00197182
HALLMARK_MYOGENESIS	163	-0.4013614	-1.7727724	0	0.00294342
**HALLMARK_TNFA_SIGNALING_VIA_NFKB**	185	-0.3829449	-1.7420073	0	0.00383526
HALLMARK_TGF_BETA_SIGNALING	52	-0.4041367	-1.4640652	0.02356021	0.03968911
HALLMARK_KRAS_SIGNALING_UP	162	-0.3229919	-1.4377202	0	0.04565698
HALLMARK_ESTROGEN_RESPONSE_LATE	183	-0.2956423	-1.3355744	0.01587302	0.10309143
HALLMARK_IL2_STAT5_SIGNALING	188	-0.2898708	-1.3152194	0.015625	0.11083192
**Positively regulated**					
**NAME**	SIZE	ES	NES	NOM p-val	FDR q-val
**HALLMARK_E2F_TARGETS**	198	0.7348152	3.1534355	0	0
**HALLMARK_G2M_CHECKPOINT**	198	0.6890323	2.9352474	0	0
HALLMARK_MYC_TARGETS_V1	197	0.6626929	2.8335598	0	0
**HALLMARK_MYC_TARGETS_V2**	58	0.7101932	2.5339458	0	0
**HALLMARK_MTORC1_SIGNALING**	198	0.57870287	2.491436	0	0
**HALLMARK_OXIDATIVE_PHOSPHORYLATION**	198	0.532552	2.2767758	0	0
HALLMARK_GLYCOLYSIS	185	0.5042823	2.1331122	0	0
HALLMARK_SPERMATOGENESIS	78	0.55520177	2.0961428	0	0
HALLMARK_MITOTIC_SPINDLE	198	0.47774455	2.049001	0	0
**HALLMARK_UNFOLDED_PROTEIN_RESPONSE**	110	0.45504516	1.8015443	0	0.00128385
**HALLMARK_DNA_REPAIR**	140	0.44054726	1.7955732	0	0.00116714
HALLMARK_INTERFERON_ALPHA_RESPONSE	89	0.44273165	1.7006303	0.0015949	0.00262745
HALLMARK_CHOLESTEROL_HOMEOSTASIS	72	0.42977247	1.5846494	0.01102362	0.00924402
HALLMARK_INTERFERON_GAMMA_RESPONSE	183	0.36937878	1.5571733	0.00144509	0.01139262
**HALLMARK_REACTIVE_OXIGEN_SPECIES_PATHWAY**	46	0.4461603	1.521564	0.01858108	0.0147807

Supplemental Table 2: Comparison of the GSEA normalized enrichment scores (NES) for patients stratified by gp78 protein. RED indicates gene sets also enriched significantly in the GSEA analysis of TCGA breast cancer patients stratified by Race (*J Clin Invest* 129, 2351-2356 (2019).

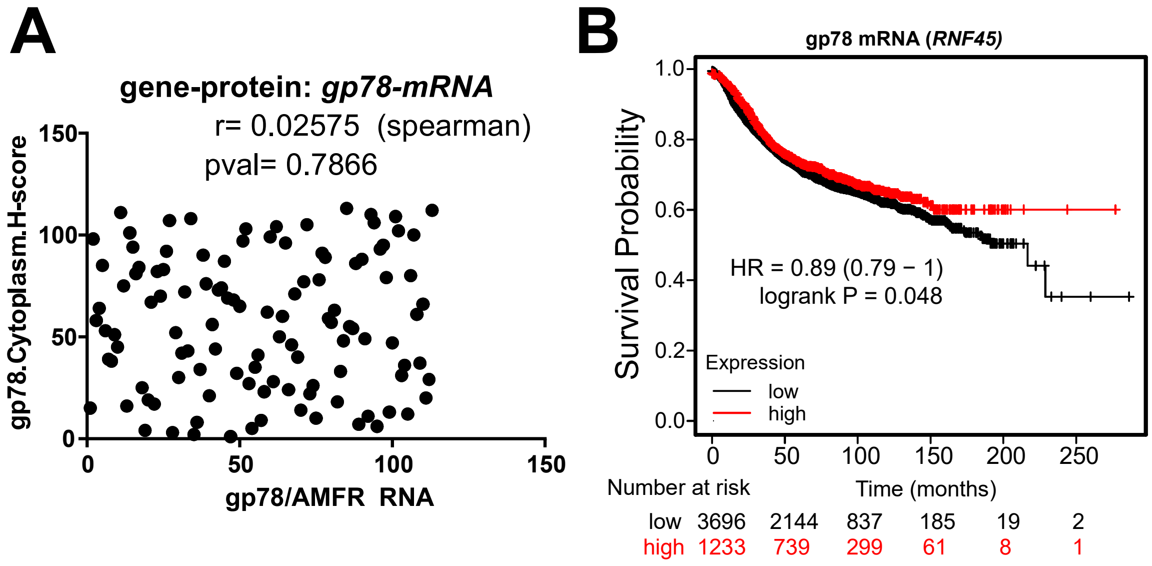

Supplementary Figure 2: (**A**) Correlation between gp78 protein expression by IHC and mRNA expression. r and p-value are shown for Spearman correlation. (**B**) Survival based on gp78 mRNA gene expression (generated from Győrffy B. *Computational and Structural Biotechnology Journal*, 2021, https://doi.org/10.1016/j.csbj.2021.07.014).

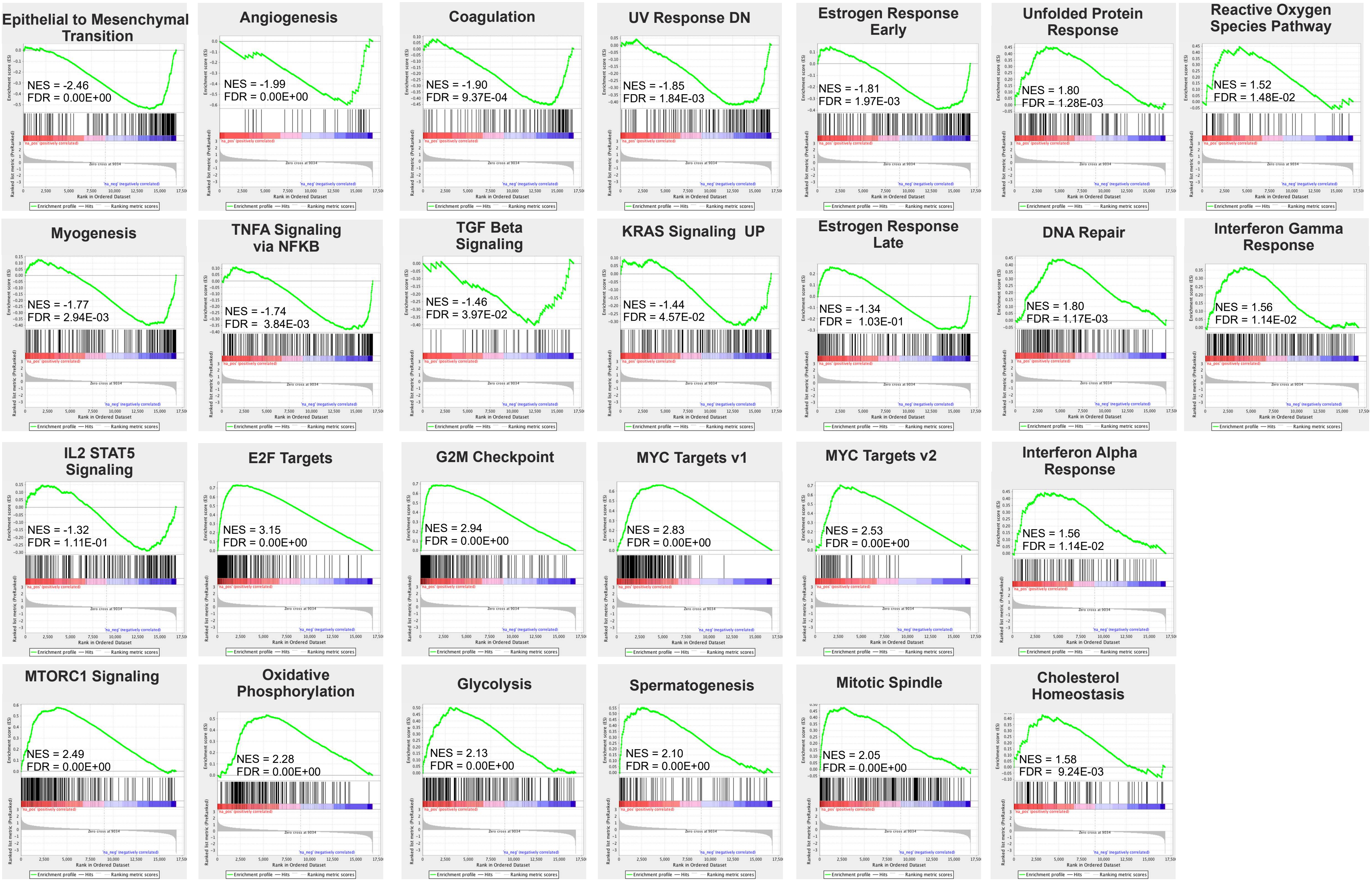

Supplementary Figure 3: Gene set enrichment analysis of RNA-seq data of ECU breast cancer patients stratified by high versus low gp78 protein levels.

A

NHB (AA) Patients GSEA					
**Hallmark Geneset Name**	**SIZE**	**ES**	**NES**	**NOM p-val**	**FDR q-val**
E2F_TARGETS	157.000	0.618	3.813	<0.001	<0.001
G2M_CHECKPOINT	133.000	0.624	3.733	<0.001	<0.001
MITOTIC_SPINDLE	80.000	0.503	2.733	<0.001	<0.001
MTORC1_SIGNALING	105.000	0.428	2.434	<0.001	<0.001
MYC_TARGETS_V1	124.000	0.404	2.411	<0.001	<0.001
GLYCOLYSIS	70.000	0.460	2.395	<0.001	<0.001
SPERMATOGENESIS	35.000	0.516	2.268	<0.001	<0.001
MYC_TARGETS_V2	41.000	0.446	2.081	<0.001	0.001
OXIDATIVE_PHOSPHORYLATION	82.000	0.339	1.824	<0.001	0.009
CHOLESTEROL_HOMEOSTASIS	28.000	0.429	**1.759**	0.007	**0.014**
UNFOLDED_PROTEIN_RESPONSE	48.000	0.353	**1.677**	0.007	**0.025**
REACTIVE_OXIGEN_SPECIES_PATHWAY	19.000	0.451	**1.660**	0.024	**0.026**

B

NHW (EA) Patients GSEA					
**Hallmark Geneset Name**	**SIZE**	**ES**	**NES**	**NOM p-val**	**FDR q-val**
E2F_TARGETS	111.000	0.470	2.749	<0.001	<0.001
G2M_CHECKPOINT	95.000	0.477	2.704	<0.001	<0.001
INTERFERON_GAMMA_RESPONSE	49.000	0.500	**2.441**	<0.001	**<0.001**
INTERFERON_ALPHA_RESPONSE	35.000	0.542	**2.421**	<0.001	**<0.001**
SPERMATOGENESIS	26.000	0.510	2.139	<0.001	0.002
MYC_TARGETS_V1	101.000	0.364	2.115	<0.001	0.002
MTORC1_SIGNALING	82.000	0.377	2.052	<0.001	0.003
MITOTIC_SPINDLE	57.000	0.373	1.891	0.006	0.014
OXIDATIVE_PHOSPHORYLATION	72.000	0.344	1.860	0.002	0.015
GLYCOLYSIS	51.000	0.376	1.849	0.003	0.014
MYC_TARGETS_V2	25.000	0.415	1.682	0.015	0.047
HEME_METABOLISM	30.000	0.365	**1.553**	0.036	**0.101**

Supplemental Table 3: Comparison of the GSEA normalized enrichment (NES) scores for patients stratified by gp78 protein. NHB patients (**A**) and NHW patients (**B**). RED indicates gene sets exclusively enriched in AA patients compared to EA patients, and BLUE indicates genes exclusively enriched in EA patients compared to AA patients.

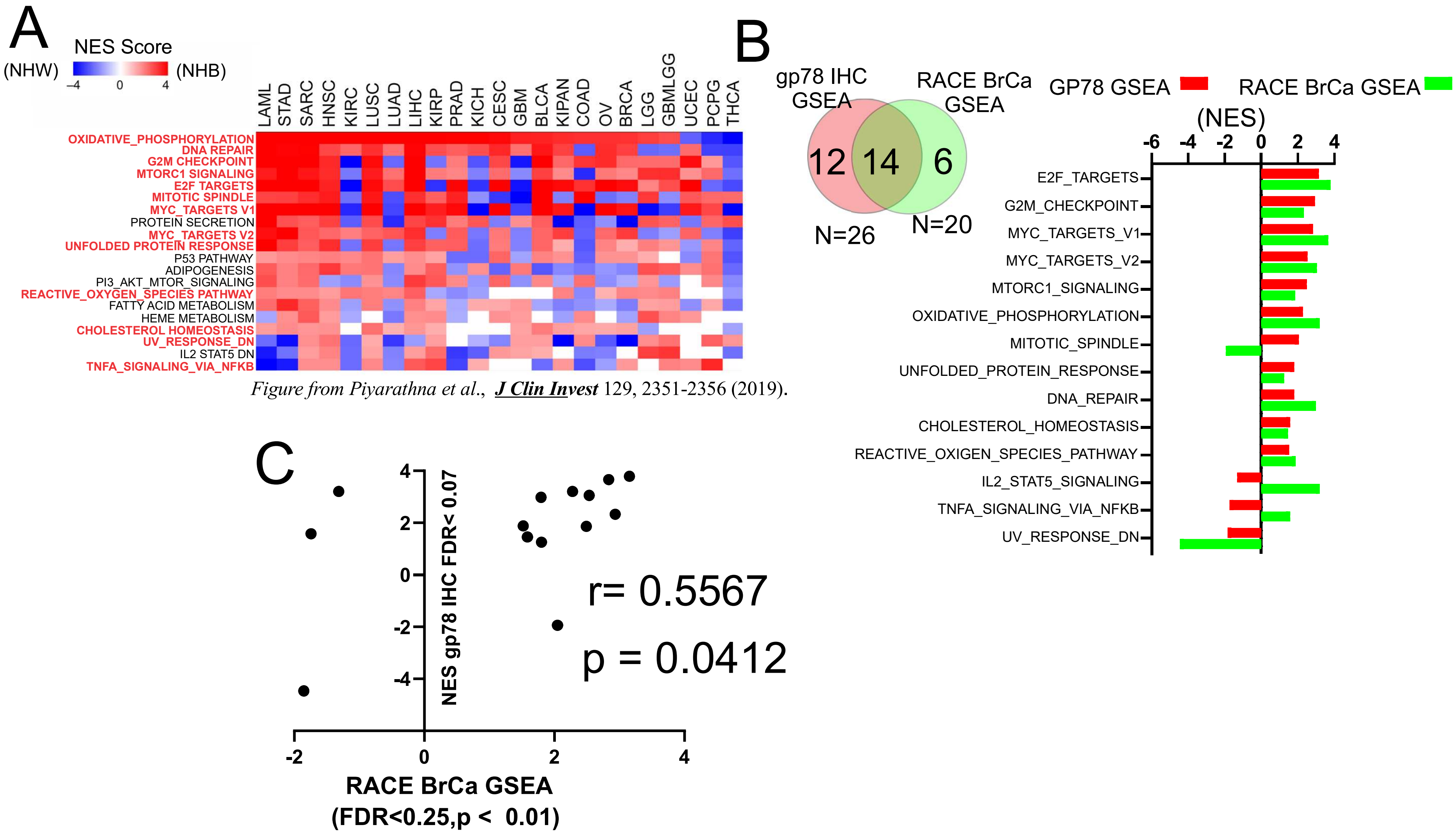

Supplemental Figure 4: Comparison of the GSEA normalized enrichment (NES) scores for patients stratified by gp78 IHC protein expression compared to the TCGA breast cancer patient tumors stratified by Race. (**A**) Heat map of normalized enrichment score of tumors stratified by race (J Clin Invest 129, 2351-2356 (2019). Gene sets in red indicated genes sets that are common to the TCGA breast cancer GSEA based on Race and the ECU breast cancer GSEA based on gp78 protein. (**B**) Venn diagram and histogram showing the overlap between gp78 protein GSEA and the TCGA breast cancer GSEA. (**C**) Spearman correlation analysis of NES derived from the TCGA breast cancer GSEA based on race and the ECU GSEA based on gp78 protein.

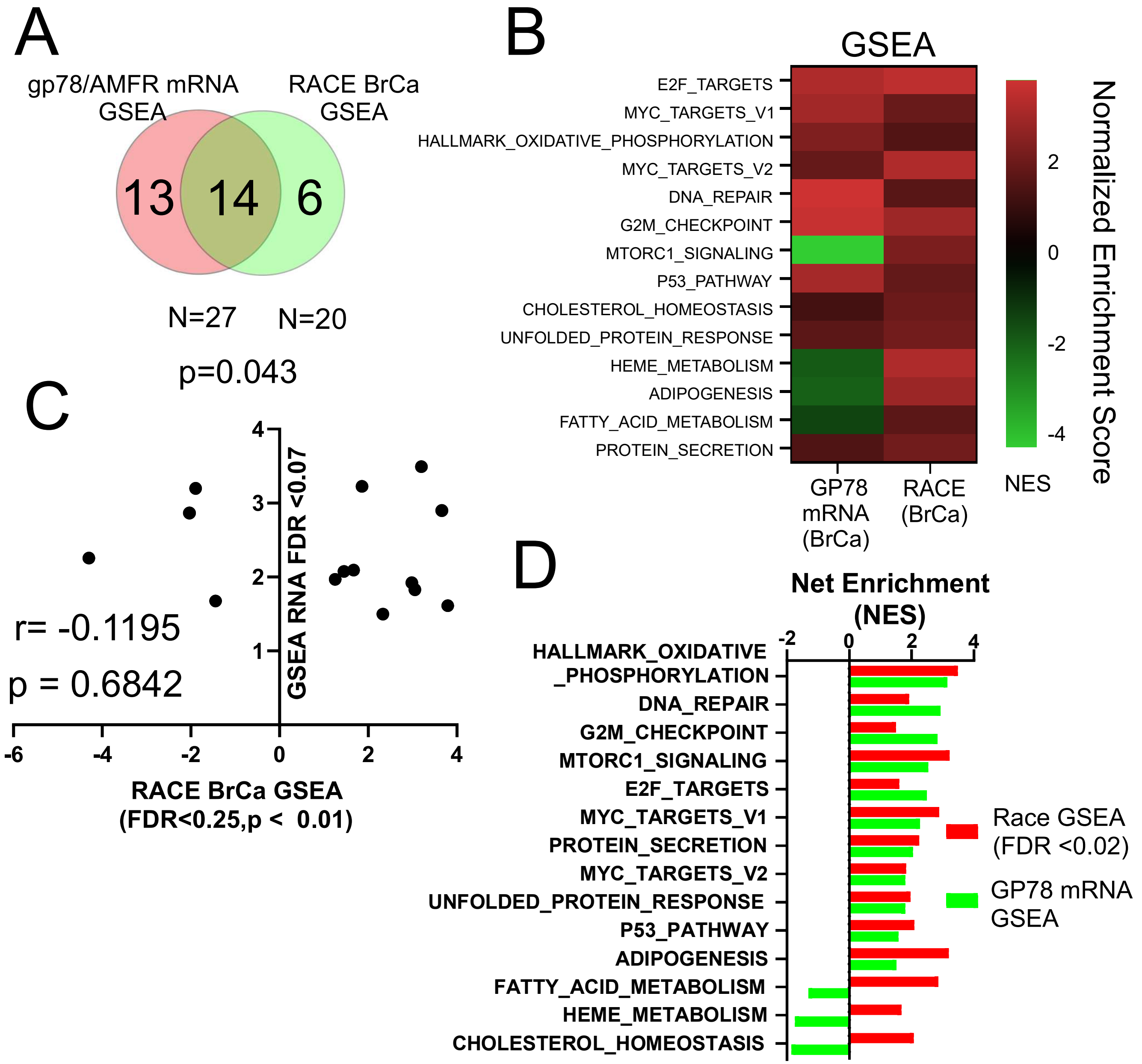

<u>Supplemental Figure 5:</u> Comparison of the GSEA normalized enrichment (NES) scores for patients stratified by gp78 RNA expression compared to RNA-seq data from TCGA breast cancer patient tumors stratified by Race. Venn diagram showing the overlap between GSEA of ECU RNA-seq data stratified by gp78 mRNA compared to TCGA breast cancer RNA-seq data stratified by Race (**A**). Heat map of TCGA and ECU breast cancer GSEA stratified by gp78 mRNA (**B**). Spearman correlation analysis of GSEA NES scores from TCGA breast cancer RNA-seq data stratified by Race and the ECU RNA-seq data stratified by gp78 mRNA (**C**). Histogram comparison of GSEA NES comparing TCGA RNA-seq data stratified by race and ECU RNA-seq data stratified by gp78 mRNA.

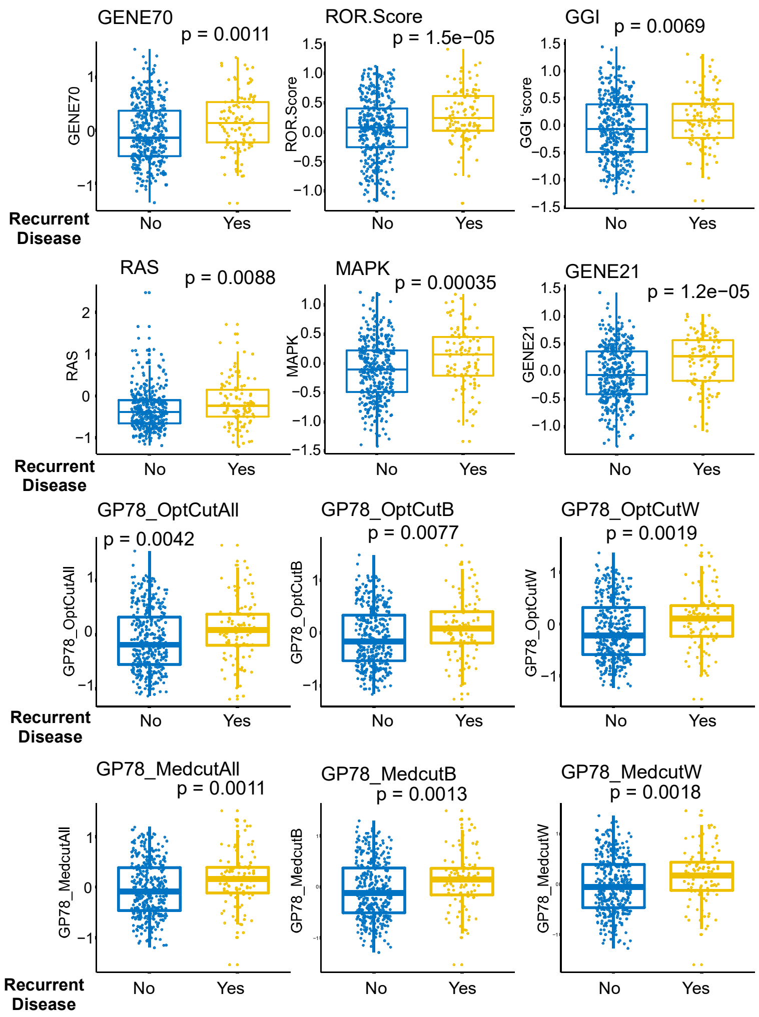

Supplementary Figure 6: gp78-derived gene module scores in patients with and without recurrent disease compared to previously established gene signatures scores (*JCO 30 (16):1966-2004* (2012)).

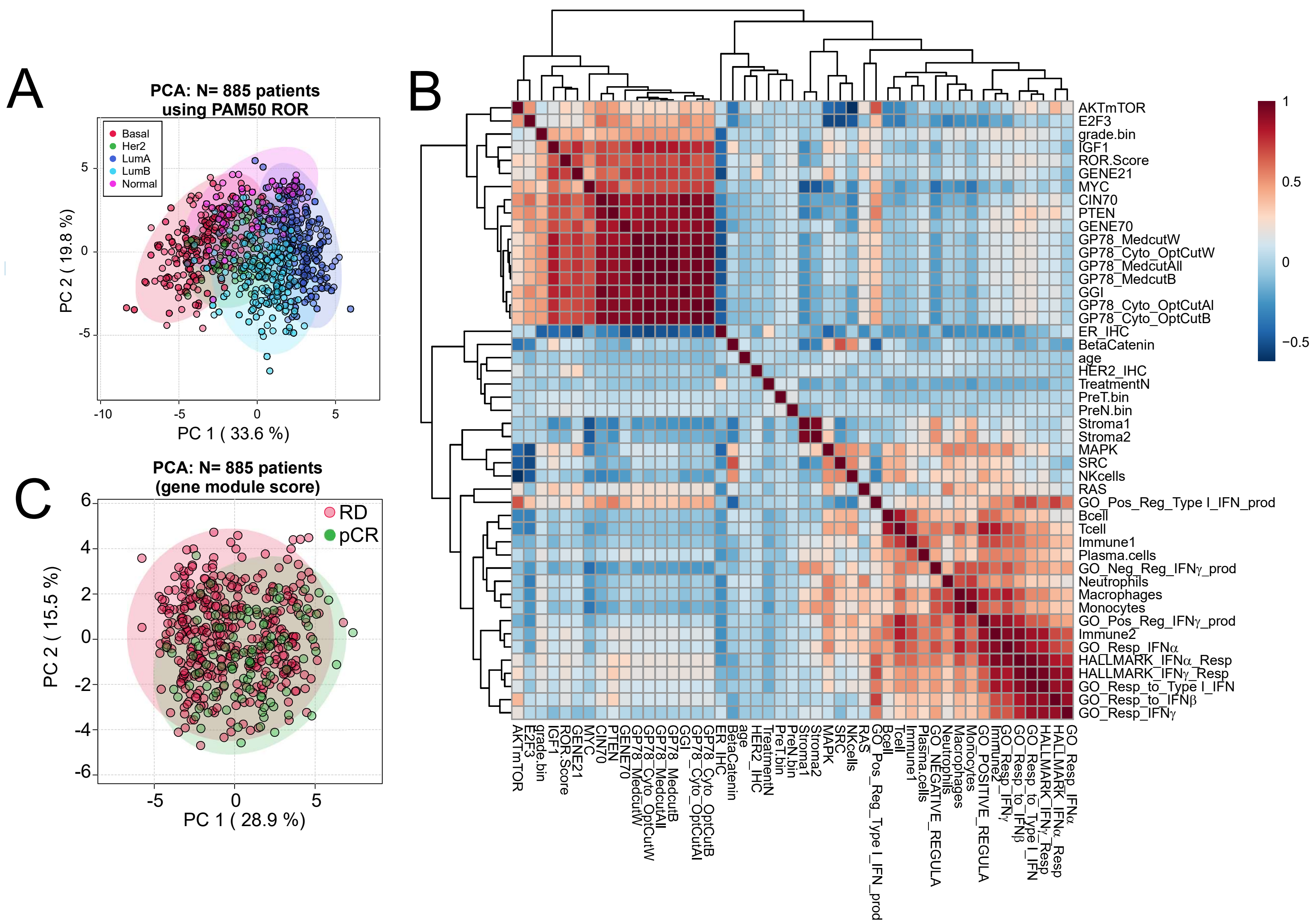

<u>Supplementary Figure 7</u>: gp78-derived gene module scores in patients with and without recurrent disease compared to previously established gene signatures scores (*JCO 30 (16):1966-2004* (2012)). (**A**) PCA analysis of patients using the PAM50 ROR gene signature. (**B**) Hierarchical clustering of correlation coefficients of gp78-derived scores and those previously described (JCO 30 (16):1966-2004 (2012). (**C**) PCA separation of all gene modules scores of patients with a complete pathological response (pCR) compared to residual disease (RD) (data taken from (*JCO 30 (16):1966-2004* (2012)).

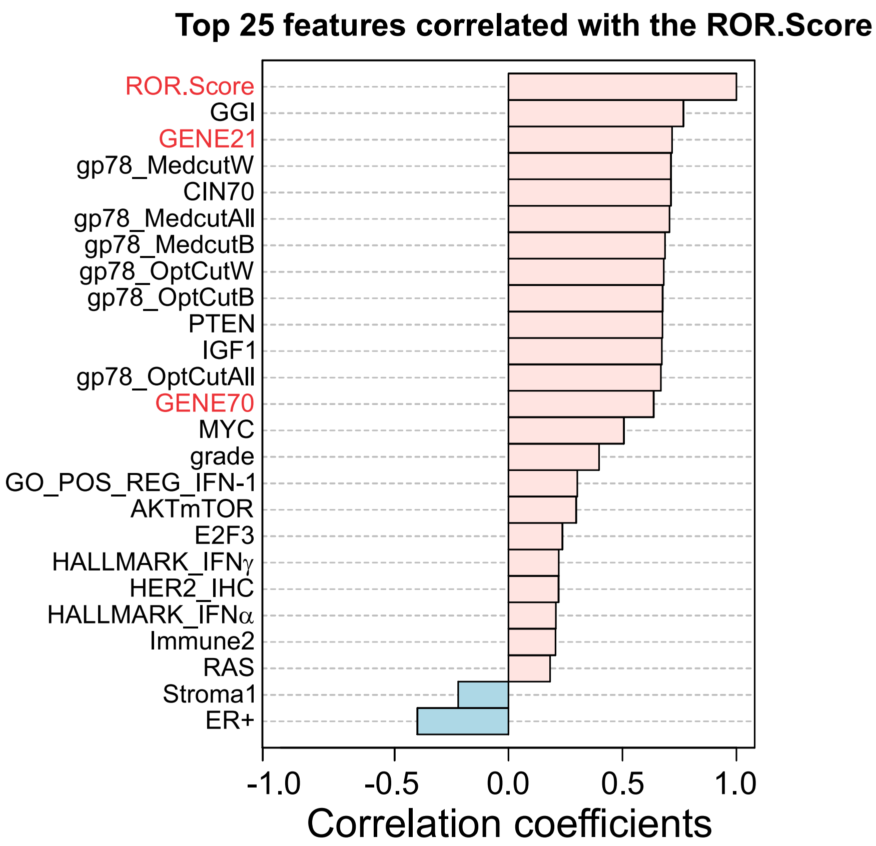

<u>Supplementary Figure 8:</u> gp78-derived gene module scores correlation with the ROR.score. gp78-derived module scores show a significant correlation with ROR (Prosignia), GENE21 (Oncotype), and GENE70 (Mammaprint) gene signatures.

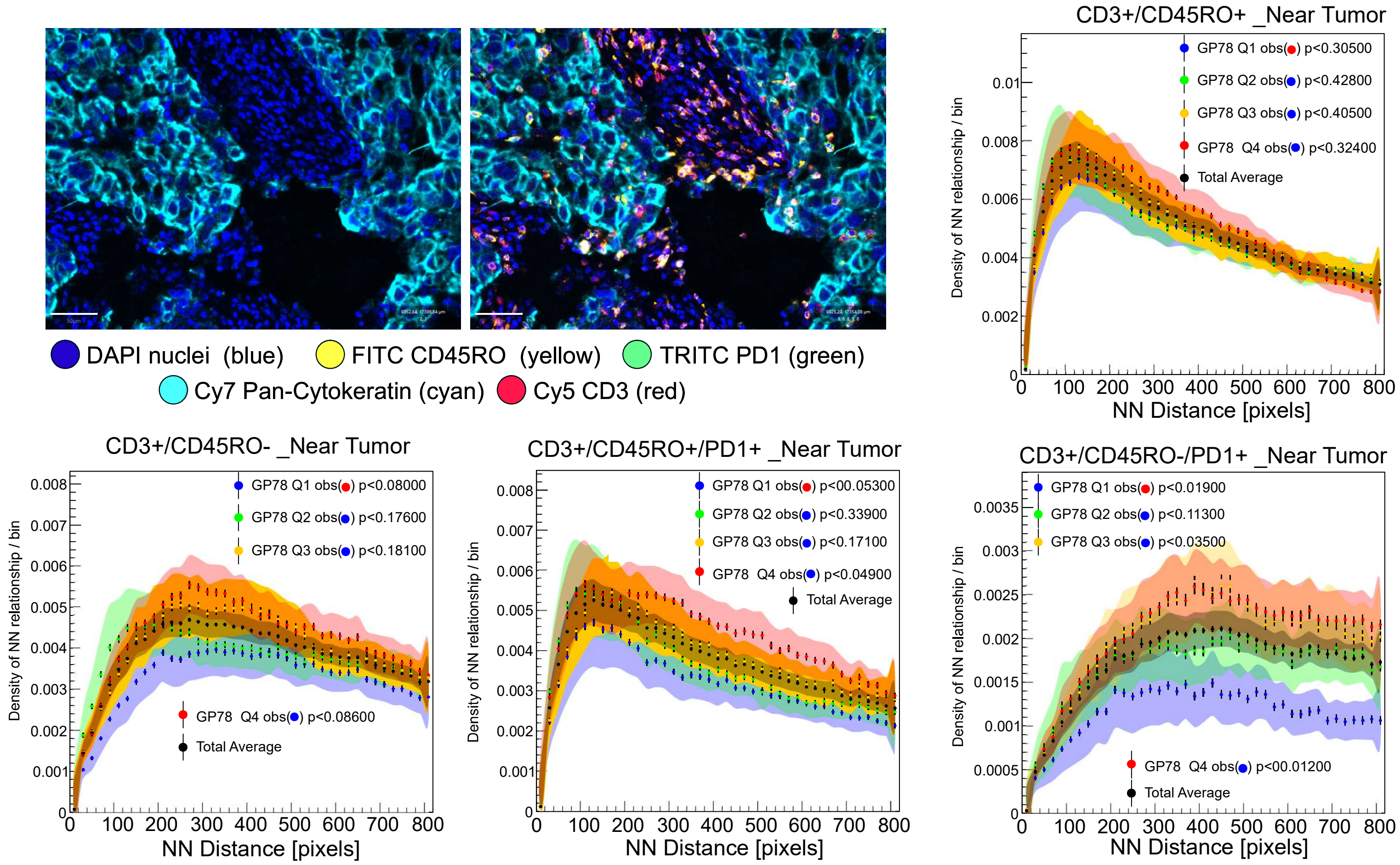

<u>**Supplementary Figure 9:**</u> Proximity analysis of the association between gp78 protein expression and immune-modulatory features in the tumor microenvironment using markers for activated memory T-cells (CD3, CD45RO) and immune exhaustion (PD-1). Data show there is no significant correlation between gp78 protein levels and T-cell exhaustion in proximity to the tumor.

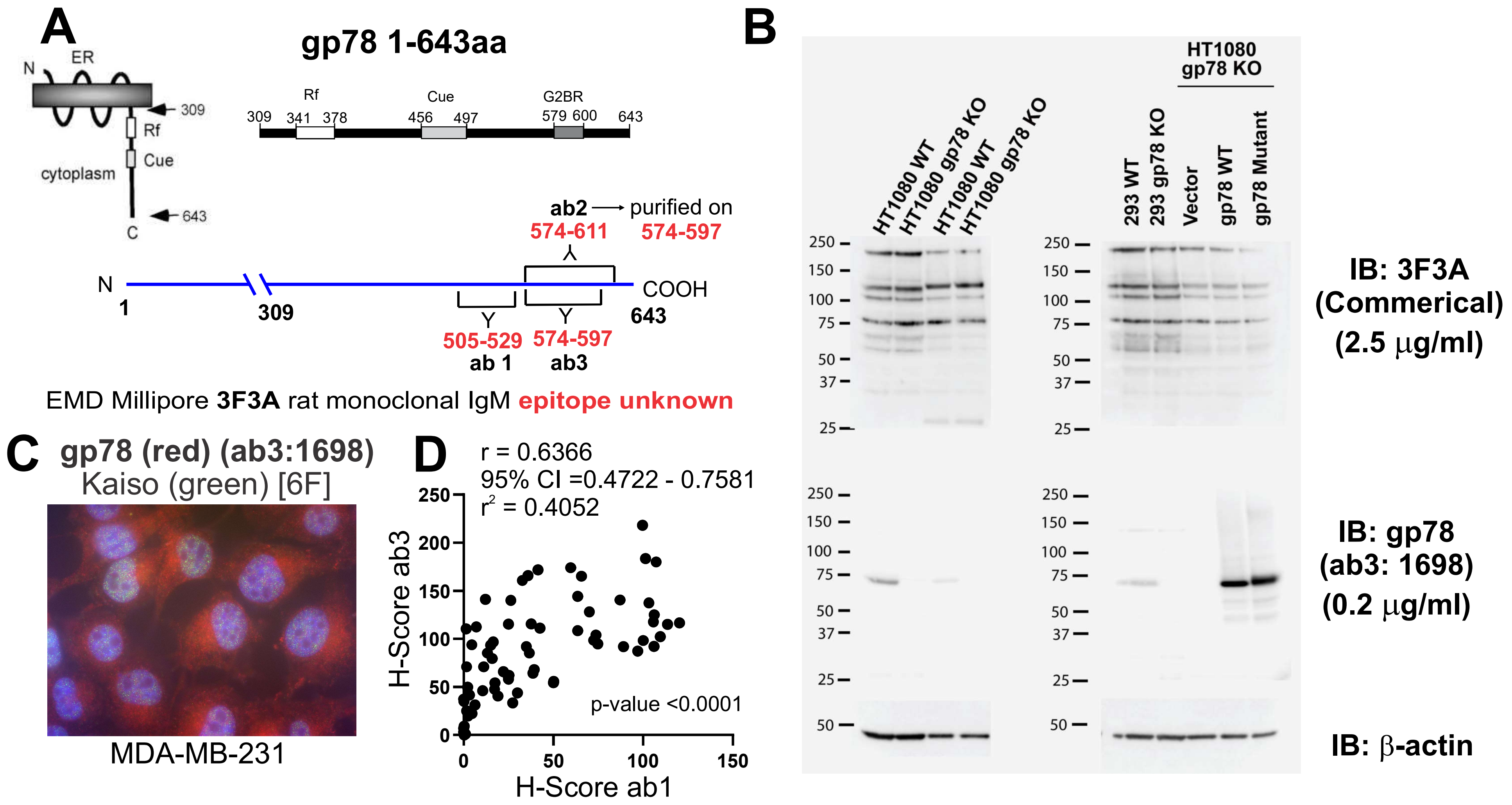

<u>Supplementary Figure 10:</u> (**A**) Schematic diagram of endoplasmic reticulum-resident gp78 showing the cytoplasmic domains. (below) is a schematic show location of peptides used as immunogens for each antibody **note that gp78 epitopes reactive with 3F3A are unknown** (**B**). Lysates from HT1080 parental and gp78 KO cells (left) and gp78KO cells rescued by wildtype and mutant gp78 were resolved by SDS-PAGE and immunoblotted with 3F3A monoclonal antibody (5 g/mL) (top). The membrane was then reblotted with gp78 ab3 (0.2 g/mL) (Middle) and then (Bottom) reblotted for actin as a loading control. (bottom). (**C**) Immunofluorescence using gp78 ab3 (1:1000) (RED) on MDA-MB-231 cells grown in culture. Blue Dapi, Green Kaiso antibody. (**D**) Correlation between IHC gp78 scores using a previously published antibody (Ab1; PMID: 16407162) compared to Ab3. Spearman R is shown.

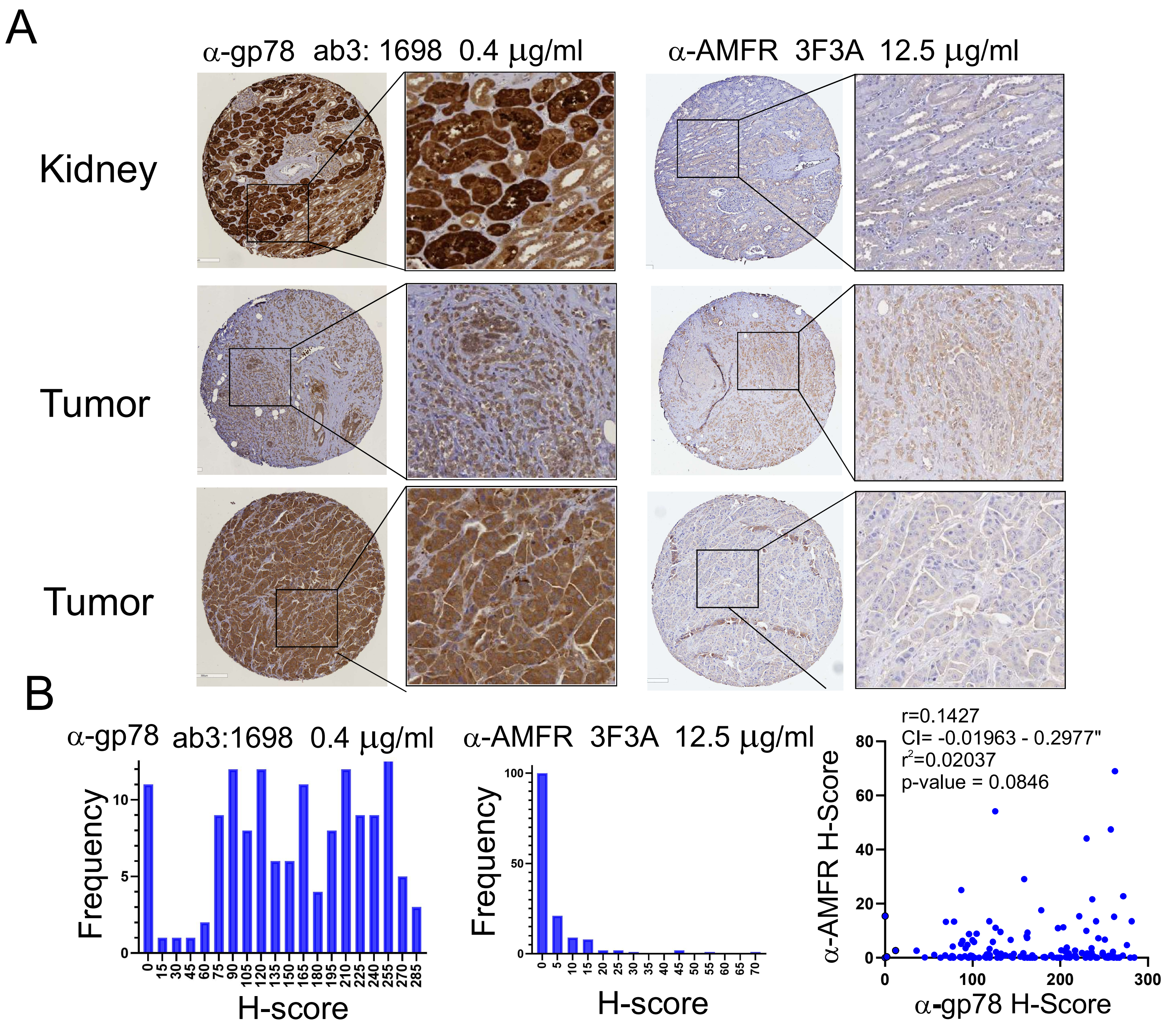

Supplementary Figure 11: (**A**) Direct side-by-side quantitative comparison of reactivity of. anti-gp78 Ab3 to 3F3A on normal tissue and tumor. (**B**) Quantitative assessment of gp78 abundance comparing Ab3 with 3F3A, including correlation of scores from a patient TMA.

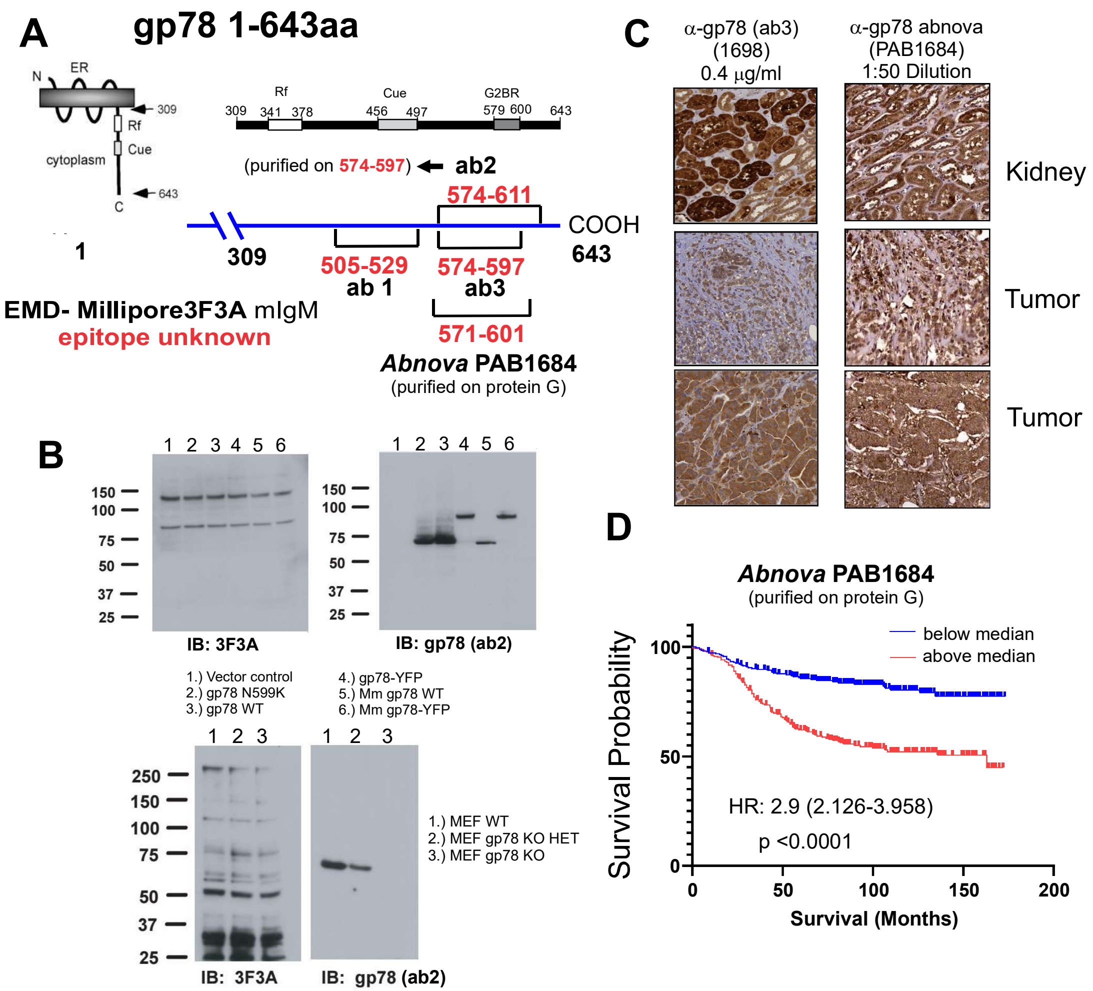

<u>Supplementary Figure 12:</u> (**A**) Schematic diagram of endoplasmic reticulum-resident gp78 showing the cytoplasmic domains. (below) is a schematic show location of peptides used as immunogens for each antibody **note that gp78 epitopes reactive with 3F3A are unknown** (**B**) <u>Upper Panel:</u> (Left) Lysates from HEK293 cells transfected with the indicated plasmids were resolved by SDS-PAGE and immunoblotted with 5 ug/ml 3F3A monoclonal antibody. N599K eliminates the putative N-glycosylation site but does not result in any apparent mobility shift. (Right) A replicate membrane was immunoblotted with gp78 ab2 (PMID:18037895 (0.2 ug/ml). <u>Lower Panel:</u> (left) Lysates from wild-type (WT; gp78+/+), heterozygous (HET; gp78+/-) and knockout (KO; gp78-/-) primary mouse embryonic fibroblasts (MEFs) were resolved by SDS-PAGE and immunoblotted with 3F3A monoclonal antibody. (Right) A replicate membrane was immunoblotted with gp78 ab2 (0.2 g/mL). (**C**)IHC profiling of kidney and tumor comparing Ab3 and commercial antibody Abnova PAB1684. **note high Abnova PAB1684 background** (**D**) Survival curve showing that that high gp78 expression based on Abnova PAB1684 predicts poor breast cancer survival.

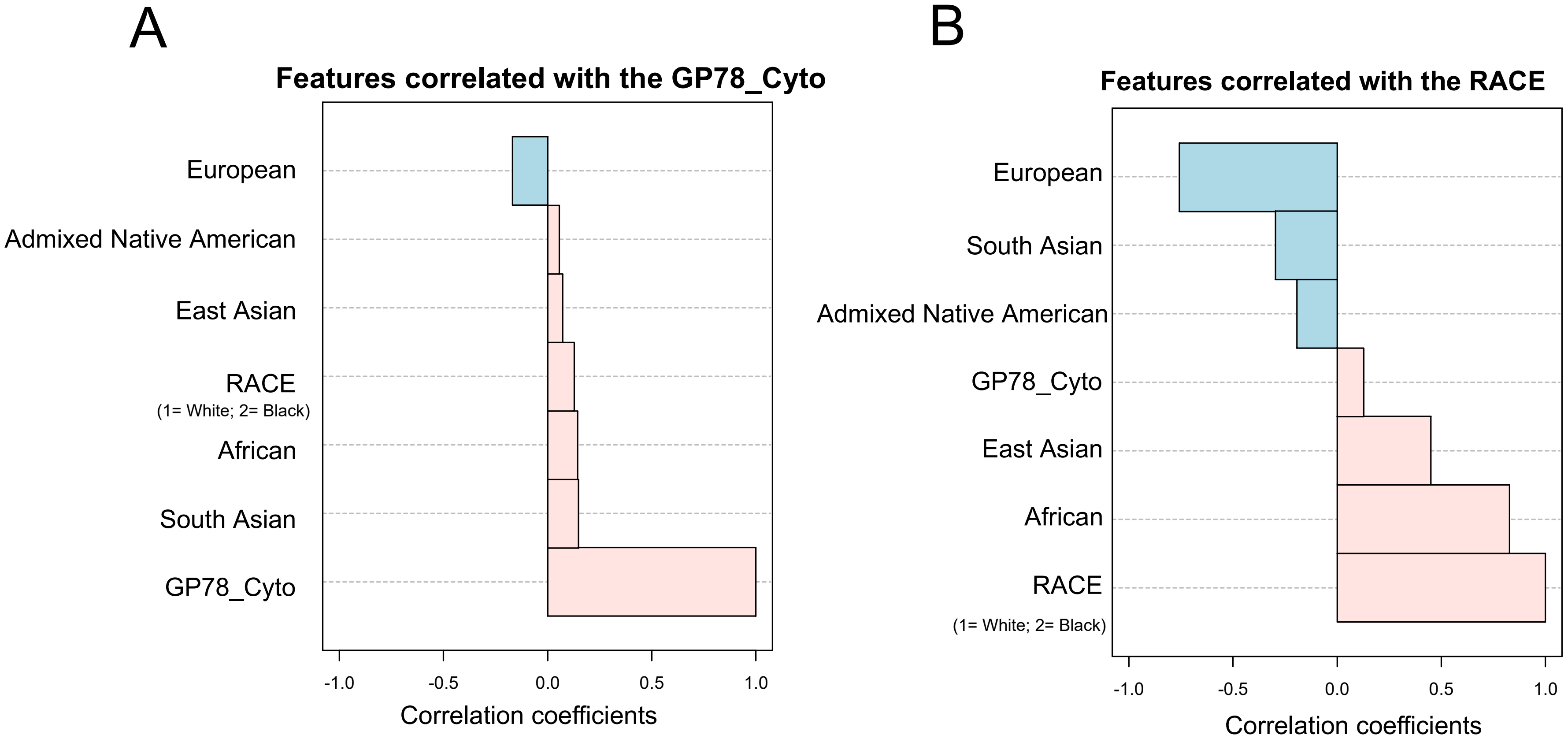

<u>Supplementary Figure 13:</u> (**A**) Correlation between genetic ancestry, race and gp78 protein scores. (**B**) Correlation between Race (white vs Black) and genetic ancestry and gp78 protein scores. RACE= self-identified Race.
